# In-hive Pesticide Exposome: Assessing risks to migratory honey bees from in-hive pesticide contamination in the Eastern United States

**DOI:** 10.1038/srep33207

**Published:** 2016-09-15

**Authors:** Kirsten S. Traynor, Jeffery S. Pettis, David R. Tarpy, Christopher A. Mullin, James L. Frazier, Maryann Frazier, Dennis vanEngelsdorp

**Affiliations:** 1 Department of Entomology, Plant Science Building, University of Maryland, MD 20742, United States; 2 USDA–ARS Bee Research Laboratory, Bldg. 306 BARC-E, Beltsville, MD 20705, United States; 3 Department of Entomology, Campus Box 7613, North Carolina State University, Raleigh, NC 27695-7613, United States; 4 Department of Entomology, Center for Pollinator Research, The Pennsylvania State University, University Park, PA 16802, United States

## Abstract

This study measured part of the in-hive pesticide exposome by analyzing residues from live in-hive bees, stored pollen, and wax in migratory colonies over time and compared exposure to colony health. We summarized the pesticide burden using three different additive methods: (1) the hazard quotient (HQ), an estimate of pesticide exposure risk, (2) the total number of pesticide residues, and (3) the number of relevant residues. Despite being simplistic, these models attempt to summarize potential risk from multiple contaminations in real-world contexts. Colonies performing pollination services were subject to increased pesticide exposure compared to honey-production and holding yards. We found clear links between an increase in the total number of products in wax and colony mortality. In particular, we found that fungicides with particular modes of action increased disproportionally in wax within colonies that died. The occurrence of queen events, a significant risk factor for colony health and productivity, was positively associated with all three proxies of pesticide exposure. While our exposome summation models do not fully capture the complexities of pesticide exposure, they nonetheless help elucidate their risks to colony health. Implementing and improving such models can help identify potential pesticide risks, permitting preventative actions to improve pollinator health.

The impact of pesticides on pollinators remains an ongoing concern, especially as new research highlights the complex web of pesticide residues entering the internal hive environment and colony food stream^1^,^2^. Honey bees (*Apis mellifera)* provide critical pollination services valued at over $200 billion worldwide^3^ and $17 billion in the US^4^. Despite increased attention, colony losses have remained elevated since 2006 in the US^5^,^6^,^7^,^8^,^9^,^10^,^11^ and other countries have reported losses as well^12^,^13^,^14^. Honey bee colonies have been proposed as terrestrial biomonitors^15^,^16^ because workers from the same colony typically forage up to 6 km away (with max distances reported as 13.5 km^17^) from the hive^18^, encompassing over 100 km^2^, and return with accumulated contaminants to the hive. A single colony, therefore, can act as a terrestrial sentinel, expanding radially into the surrounding environment to collect food resources and acquiring contaminants including pesticide-laced pollen that is then stored in the colony as “beebread.”

Commercial beekeepers have increasingly struggled with high rates of colony morbidity and mortality, in part because of increased pressure of the parasitic varroa mite (*Varroa destructor*)^9^,^10^,^19^, pathogens (primarily viruses and bacterial infections)^20^, pesticide contamination of hive matrices^21^,^22^, poor nutrition^23^,^24^,^25^, and frequent queen losses^26^. Human health exposure science has called for a top-down approach to understanding environmental causes of disease by examining changes to the body’s internal chemical environment over time from chemical contaminants (referred to as the exposome, the totality of exposures received during life^27^), instead of a bottom-up approach that focuses on the impact of external pollutants in water and air^28^. In this study, we apply this concept to honey bee health, measuring the pesticide exposure within the internal hive environment during the entire beekeeping season, and relating these measures to colony health outcomes.

Commercial operations that provide pollination services and produce high volumes of honey regularly move their colonies among crops for pollination, natural areas for honey production, and intermediate “holding” yards. These three different colony settings impose diverse exposure risks to the internal colony environment from agricultural pesticides and beekeeper-applied varroacides. Holding yards frequently increase colony density, potentially facilitating the greater exchange of disease and parasites as well as increased nutritional stress if colonies compete for finite food resources. Management practices also vary among operations, with some beekeepers managing colonies on an individual colony basis while others manage on a per-apiary basis, frequently equalizing colony strength by exchanging combs of brood among colonies.

Understanding and quantifying the risks of pesticides entering the hive environment is difficult and expensive^29^,^30^. Pesticide risk is currently determined via short-term acute contact and oral toxicity tests on adult bees (i.e., LD_50_ levels), which are devoid of synergistic, cumulative, and sublethal effects on the colony. Chronic toxicity tests over 10 days are suggested, but the standardized tests for larvae and adult bees are still in development by the Organization for Economic Co-operation (OECD). Relatively little is known about the effects of multiple pesticide residues, their synergistic interactions, and the effects on different honey bee life stages or wild pollinators. Like most other insects, honey bees rely on detoxification enzymes, primarily the cytochrome P450s, to metabolize synthetic chemicals. The honey bee genome^31^ revealed a paucity of encoded P450s compared to other insects^32^, suggesting this reduced diversity in enzymes may contribute to an increased sensitivity to pesticides. Studies indicate that honey bees may rely on a small number of enzymes to detoxify both natural and synthetic xenobiotics^33^, increasing the risk that exposure to multiple pesticides in different matrices (e.g., pollen and wax) may overwhelm their detoxification system as the residues overwhelm available receptor sites. This effect is likely more pronounced if multiple pesticides have the same mode of action (MOA)^2^.

Bees are evolutionarily adapted to feed on pollen^34^, which honey bee foragers collect from plants and store in cells adjacent to the brood (developing young). Nurse bees consume and convert the beebread into proteinaceous glandular secretions fed to developing larvae^35^; nurse bees can consume over 100 mg of beebread during the 10–12 day window when these bees activate their food producing glands and feed larvae^36^,^37^. Consumption is greater (estimated at 240 mg) in long-lived winter bees, which consume ~2 mg of pollen per day for general hive maintenance and can live over 120 days^37^,^38^.

In a previous study^26^, we documented the prevalence of easy-to-measure putative risk factors (e.g., prevalence and abundance of pathogens and parasites, overt brood diseases) in colonies managed by three commercial beekeepers pollinating crops along the east coast of the United States over a beekeeping season. Further, we linked the incidence of putative risk factors with colony mortality. In so doing, we observed two factors correlated with 3x greater colony mortality than non-symptomatic colonies, 1) colonies that experienced a “queen event”, where the established queen had been or was in the process of being replaced, or 2) an unusual brood disease condition termed Idiopathic brood disease syndrome (IBDS).

In this study, samples from various colony matrices (beeswax brood comb, adult bees, and beebread) were collected for pesticide analysis. These samples were collected to document any changes in pesticide burden in colonies over time. Limited resources for pesticide analysis permitted us to only explore possible relationships between colony mortality and pesticide loads in beebread over time and to determine the impacts of pesticides in wax at the beginning and end of the study between colonies that varied in colony mortality and queen events. We summarize the overall pesticide burden using three different models: a Hazard Quotient (HQ)^30^ model, the total number of different pesticide products, and the total number of relevant pesticide products that contribute at least 0.5% of a bee’s LD_50_. All three models attempt to illustrate a simplified risk to bees from consuming contaminated food or living in a pesticide-rich environment. Using these three models, we describe the pesticide in-hive exposome experienced by bees, through contact with wax comb and consumption of beebread, in colonies during an entire beekeeping season as they were moved into different crops. Using the number of products and HQ model that encompasses contact and oral risk from multiple residues detected in different hive matrixes, we elucidate relationships among individual pesticides, categories of pesticides (insecticides, varroacides, fungicides, and herbicides), pesticide MOA, and colony morbidity and mortality. This approach is an attempt to model the role that real-world pesticide exposure may have on colony health using admittedly oversimplified, additive models. While this approach makes certain assumptions and simplifications (see below), it is a first step toward evaluating the impacts of multiple residues in the colony environment on colony health.

## Methods

**Figure 1:**
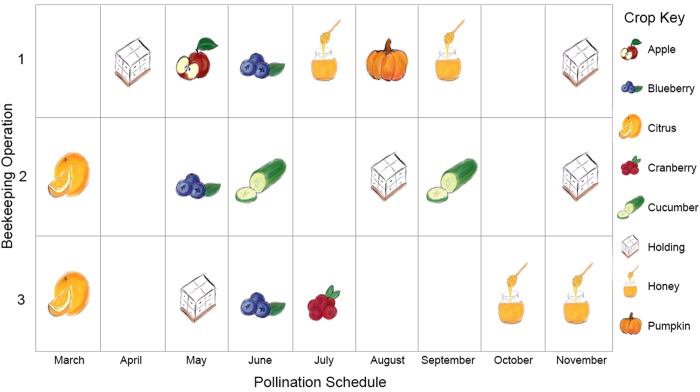
Pollination schedule of the three commercial beekeeping operations tracked over time. Multiple bee bread samples were collected at each of the above time points for pesticide analysis. At the end of the study, samples for each time point were retrospectively pooled from 3–4 colonies within the same operation that perished at similar times or survived the season. For each available time point, samples were always pooled from the same 3–4 colonies and each time point was represented by at least four pooled samples.

A study was conducted to monitor the morbidity and mortality of honey bee colonies owned by three different migratory beekeepers moving colonies up the east coast of the United States^26^. Colonies were monitored for approximately 300 days between March 2007 and January 2008. In addition to the 72 established and overwintered colonies monitored and reported in vanEngelsdorp *et al.*^26^, 19 colonies started from packages in February of 2007 were also monitored. The package colonies were run and operated by OP1 bringing the total colonies monitored for this current study to 91 (OP1: n = 39, OP2: n = 24, OP3: n = 18). We began monitoring colonies in Florida and inspected surviving colonies as they moved up the east coast to pollinate various crops (Fig. 1). By January 2008, all surviving colonies had been moved back to Florida. Colonies were inspected each time they were moved to a new location. In addition to monitoring hive conditions (as outlined in vanEngelsdorp *et al.*^26^), samples of bees, wax, and beebread were removed from colonies at each inspection. Details of the migratory routes of each operation, as well as the timing of different colony sampling and assessments, are presented in vanEngelsdorp *et al.*^26^, but a visual overview of the surveillance effort is also summarized in Fig. 1. Samples collected for pesticide analysis were collected from each colony at the time of colony health inspection.

Bees: Samples of adult bees (~200) were collected from brood frames from individual colonies at each inspection period and placed in 50 ml tubes and stored on dry ice, then transferred to freezers (−80 °C) until processed. We analyzed a subset of these bees (n = 38), collected at the start of the study. However, because of the low rate of residue detection in these bees and limited resources, we did not analyze any other adult bee samples.

Wax: a ~5 g sample of comb (wax) was collected from individual hives at each inspection. Whenever possible, samples were collected from an area of used brood comb that did not contain any beebread, honey, or brood. These samples were collected and stored on dry ice, then transferred into a freezer for long-term storage. Because OP2 managed their hives at the apiary level (which involves frequent equalizing of combs among colonies) rather than at the individual colony level (which does not), no wax samples from their operation were analyzed. Because of limited funds, we could only analyze a subset (n = 108) of all wax samples; we analyzed the wax samples of the first inspection period and a sample collected from the last time that colony was inspected while still alive.

Beebread: Disposable wooden stir sticks were used, one per colony, to remove beebread from several cells of comb. Removed beebread was placed in 1.5 ml Eppendorf tubes and immediately stored on dry ice, then transferred to a freezer (−20 °C) until processing. Because of limited resources for sample processing and limited beebread availability, beebread samples collected on the same date were retroactively pooled within an individual operation by taking approximately equals amounts of beebread from 3 or 4 colonies that ended up dying at the same time or surviving the entire beekeeping season, resulting in 24 pooled samples monitored over the course of the study (for a total of n = 147 samples analyzed). For specific questions, relevant subsets of these 147 samples were used to analyze impacts of pesticide exposure on survival, crop, season, and colony type. For example, to determine the impact of the early pesticide exposure from March–June on established colonies that lived or died throughout the entire beekeeping season encompasses 34 relevant samples.

Bees, wax, and pooled beebread samples weighing approximately 3 grams were measured into 50 ml plastic centrifuge tubes and sent on ice to the USDA-AMS National Science Laboratory in Gastonia NC for multi-pesticide residue analysis. Samples were extracted and analyzed for 171 pesticides and associated degradates at the part per billion (ppb) level as described in Mullin *et al.*^21^.

### Number of products and Hazard Quotient

We calculated the number of products found in each matrix and subsequently calculated the Hazard Quotient (HQ) for each matrix in each sample. The HQ was calculated using a similar method described in Stoner *et al.*^30^. Briefly, the risk of available pollen to a consuming bee was estimated by summing the dose divided by a screening benchmark; in this instance, the sum of all pesticide residue concentrations in ppb divided by their respective LD_50_ in μg/bee for each residue in a given sample. This provides an estimate of the frequency that 50% lethal dose equivalents for bees are present in the food, wax, or in bees themselves. Actual exposure from beebread depends on individual consumption rates. Residue detections are measured in μg/kg (ppb) divided by an LD_50_ in μg/bee. LD_50_ values (Table S1) represent averaged 24–72 h adult acute toxicities available from the US EPA Ecotox Database (http://cfpub.epa.gov/ecotox/); the University of Hertfordshire Pesticide Properties DataBase (PPDB, http://sitem.herts.ac.uk/aeru/ppdb/en/index.htm); and some additional primary literature^39^,^40^,^41^. For degradates with an unknown LD_50_, the LD_50_ of the respective parent compound was used in the HQ calculation. Assuming that a bee consumes at least its average body weight (~100 mg) in pollen during development^36^,^42^, then the HQ that would result in a 50% kill dose is 1,000,000 mg/100 mg = 10,000—assuming that toxic effects are cumulative, additive and not synergistic or antagonistic, and the products do not degrade or are not detoxified. However, the screening benchmark should be a no-adverse effects threshold, not a 50% mortality dose. By including a safety factor of 1/10^th^—as does the European Food Safety Authority for setting a limit of concern for bee residues^40^ or the EPA when setting food pesticide tolerances—then a HQ threshold of 1,000 would correspond with potential for some initial bee acute toxicity. A score of 1,000 corresponds to a bee consuming 1% of their LD_50_ daily, which adds up to 10% of their LD_50_ during the 10 day nursing phase. Arguably, a more robust HQ calculation would use LD_10,_ or no observable adverse effects levels (NOAEL) instead of LD_50_’s. However, these numbers are not available for all products detected^43^.

Both the number of products and HQ models are additive and over simplified models to quantify pesticide exposure. For instance, they do not account for synergistic or antagonistic effects of co-occurring pesticides, and they assume that pesticides do not degrade and are not detoxified when consumed. These assumptions are unrealistic, as some products are known to synergize and honey bees detoxify foreign substances^44^,^45^. At best the HQ provides an underestimate of total exposure. Unfortunately, the current state of knowledge does not permit for the development of more robust models that include these factors, and thus we use these more simplistic models as a starting place to help understand the risk posed by the real-world exposome experienced by commercial honey bee colonies.

We used two approaches to analyze the simple number of products. For our first approach, we simply summed all products detected, even when they were found at trace amounts in a given sample. However, some pesticides were detected at such low levels that they did not contribute substantially to the HQ score. As such, for our second approach we determined if these low level residues were confounding the analysis. We considered a sample to have “relevant” pesticide loads if they had HQ scores greater than 50. At this level, the sample had a pesticide load that contributed daily at least 0.05% of a nurse bee’s LD_50_, which corresponds to 0.5% during the 10-day nursing phase. We quantified the number of different products in a given matrix (total residues) as well as the number of products that contributed at least 50 points to a given samples total HQ score (50+). We considered a pesticide level as “elevated” if the total HQ in a sample was 1,000 or more. This level, assuming the pesticide accumulates over 10 days without degradation, is equivalent to a nurse bee consuming 1.0% of an LD_50_ per day or 10% of her LD_50_ during her nursing phase. Detected products were grouped by category; as either insecticides, fungicides, herbicides or varroacides, the latter being residues of products that beekeepers use to control *Varroa*. Further, for insecticides, varroacides, and fungicides, we categorized individual products by their mode of action (MOA) (see Supplemental Table S1). The same HQ risk assessment was applied to 38 adult bee samples, 108 wax samples, and 147 pooled beebread samples. However, since residue concentrations are significantly higher in wax, and transmission routes poorly understood in this matrix, only samples with a HQ_wax_ > 5,000 were classified as elevated.

*Analyses:* HQ score, total residues and relevant residues (50+) were calculated as described above in Microsoft Excel (Microsoft Office Professional 2010, Seattle, WA). Statistical analysis was conducted using JMP^®^ Pro 11.0.0 (SAS, Cary, NC). To determine significance, we used a generalized linear model (GLM) with a Poisson or binomial distribution as necessary, and corrected for overdispersion if appropriate. Relevant model effects such as collection date, crop, environment, or operation were included, as described in the results and figures. Chi square results from these analyses are reported. In all figures, mean results are reported with S.E. indicated. Post-hoc analysis of significant differences are indicated by different letters (α = 0.05).

**General HQ, total residues, and 50+ pesticide residues:** For beebread residues in individual operations, we determined differences in HQ_bbread_ and total number of pesticide residues by crop and sampling period. For wax, we determined HQ_wax_, total pesticides, and 50+ residues with inspection period and colony survival as model effects. We then analyzed if the number of products with a given MOA had an effect on HQ_bbread_ or HQ_wax_ or varied by environment. **Colony Survival:** For wax samples, we examined the impact of total and 50+ residues on colony survival for each MOA. Further, we examined colony survival, including HQ_wax_, sampling time, and colony type as model effects. Since there was a significant effect of colony type, we repeated the analysis for established colonies and packages separately. For bee bread samples, we analyzed HQ_bbread_ for the early beekeeping season (March through June) with collection date and survival as model effects. We repeated the analysis with colony type as an additional model effect, with similar results except that colony type (established vs. package) was also highly significant. We thus analyzed the HQ_bbread_ of packages separately for the summer beekeeping season (May-August) with collection date and survival as model effects. To determine if colonies that died during the beekeeping season differed in their HQ_pesticide categories_ we included collection date as a model effect. Due to numerous samples contaminated with chlorothalonil, we analyzed if HQ_bbread_ score contributed by chlorothalonil varied by colony survival. **Imminent death:** For bee bread samples, imminent death was defined as one or more of the colonies in a pool perishing within one month. We analyzed the impact of HQ_bbread_, total and 50+ residues with collection date and imminent death as effects. To determine if HQ_fung_ varied with imminent death during the summer season (May-Aug) we included collection date and imminent death as model effects. We repeated the analysis, substituting crop for collection date. **Queen events:** For wax samples, we analyzed the impact of queen events, with collection order (first/last) and HQ_wax_, total or 50+ residues as model effects. We repeated the analysis to determine if number of products with a given MOA had an impact on queen events.

## Results

### Pesticides: Prevalence and HQ risk

A total of 93 different pesticide residues were identified in the analyzed samples (Table S1). A total of 13, 61, and 70 different pesticides and/or residuals were found in bees, beebread, and wax respectively (Tables S2, S3 and S4).

Bees: The maximum number of pesticides found in a single bee sample was 4. Five samples were pesticide-free, while all other bee samples (n = 33) had at least one product detected. Few pesticide residues were detected per bee sample (1.39 ± 0.15), with the main contributors being beekeeper-applied varroacides, coumaphos and fluvalinate, which were detected in 23.7% and 81.6% of samples, respectively (Table S2). Across all samples, only 1 of 38 live bee samples had an elevated HQ_bee_ (HQ_bee_ = 1,256), primarily a result of contamination with fipronil detected at 9.9 ppb. With its low LD_50_, fipronil contributed 1,222 points to the HQ_bee_. All other samples had an HQ_bee_ < 50.

Beebread: Samples were retrospectively pooled from 3–4 colonies that perished at similar times, so that pesticide exposure at multiple time points during the season could be evaluated. The maximum number of different pesticide products found in a pooled beebread sample was 20. All samples (n = 147) had at least one product detected. On average, each pooled sample had 7.22 ± 0.30 different pesticides or metabolites. Across all pooled beebread samples, 1,061 pesticides and their metabolites were detected (see Table S3, Fig. S1); however, of these only 14.7% (n = 156) contributed at least 50 points to the HQ_bbread_ score and were considered “relevant” (Table S3, Fig. S1). Seventeen individual pesticide detections contributed more than 1,000 points to the HQ_bbread_ (chlorpyrifos = 9; fenpropathrin = 5; fipronil = 1; pyridaben = 2). Insecticides were most commonly detected (n = 363; 34.3%), followed by varroacides (n = 343, 32.3%), fungicides (n = 204, 19.2%), and lastly herbicides (n = 151, 14.2%). The average HQ_bbread_ for all samples was 445 ± 62.8. The total HQ_bbread_ score was greater than 1,000 in 15% of samples (n = 22). Insecticides were the largest contributor to the total HQ_bbread_ score (85.9%) across all samples, followed by varroacides (10.6%), fungicides (3.5%), and herbicides that contributed only minimally (<0.01%). Fungicides were very common, appearing in 70.1% of all beebread samples (n = 103). In the current study, fungicide residues were detected 77 times at over 100 ppb (Table S5), over 2.5x more frequently than insecticides. All fungicides that contribute 5 points to the HQ_bbread_ score were found at more than 100 ppb. The majority (91.2%) contributed less than 50 points to the total HQ_bbread_, generally because fungicides have relatively high LD_50_s that ranged from 25-2,430 ppb. Multiple fungicides in a single sample occurred frequently, with 44.9% (n = 66) of all samples tested having two or more fungicide residues (Fig. S2).

When grouped by their MOA, we found that HQ_bbread_ scores were significantly elevated when more than two insecticides from group 1 (1.AChE, acetylcholinesterase inhibiting) occurred in the beebread sample (χ^2^ = 53.56, df = 5, n = 147, p < 0.0001). Samples with zero or one group 1 insecticide had a mean HQ_bbread_ = 139.7 ± 28.7, while samples with 2 + products had a mean HQ_bbread_ = 810.1 ± 119.5, ranging from 579.1 ± 140.7 to 895.1 ± 6167.4 depending on the number of products detected. One commonly detected varroacide, the organophosphate coumaphos, has a group 1 insecticide MOA; this product contributed 11.6% to the average HQ_bbread_ score of 228.9 contributed by this MOA group. Group 2 insecticides (2.GABA, GABA-gated chloride channel blockers) had a similar impact (χ^2^ = 16.29, df = 4, n = 147, p = 0.0026), with HQ_bbread_ scores significantly lower when no group 2 insecticides were detected in beebread (mean HQ_bbread_ = 280.9 ± 71.3) compared to when group 2 products were present (mean HQ_bbread_ = 369.4 ± 99.88 to 3,825.9 ± 0). No varroacides had a group 2 MOA.

Wax: The maximum number of residues detected in a single wax sample was 39, with a mean of 10.17 ± 0.47 per sample. Of the 108 samples analyzed, all had at least three products detected. Altogether, 1,108 pesticide residues were detected, of which 32.3% (n = 358) contributed at least 50 points to the HQ_wax_ and were considered relevant (Table S6). Total HQ_wax_ was significantly lower in samples taken at the start of the beekeeping season compared to samples taken at the last inspection period (Fig. S3, χ^2^ = 5.50, df = 1, n = 108, p = 0.019), regardless of colony survival (χ^2^ = 0.024, df = 1, n = 108, p = 0.8751). Total HQ_wax_ was above 1,000 for 77.98% (n = 85) and above 5,000 for 7.34% (n = 8) of wax samples. Residues that contributed more than 1,000 points to the HQ_wax_ score included the varroacides coumaphos, fluvalinate, and the amitraz breakdown product DMPF, as well as the insecticides deltamethrin, fenpropathrin, fipronil, and permethrin (see Table S6). While never exceeding the 1,000 HQ_wax_ threshold (largely on account of its high LD_50_), the fungicide chlorothalonil was present in 68.8% of wax samples (n = 75) with a mean concentration of 1,635.0 ± 756.9 ppb, making it the most abundant wax contaminate after varroacides (see Table S6). In one wax sample, chlorothalonil levels were detected at 53,700 ppb, a higher concentration than any of the beekeeper applied varroacides, and contributed 483.8 points to the HQ_wax_ of that particular sample. Low amounts of neonicotinoid insecticides (group 4, 4.nAChR, nicotinic acetylcholine receptor competitive modulators) were found in six wax samples; two were contaminated with imidacloprid at 2.4 and 13.6 ppb, contributing 60.3 and 341.7 points to the HQ_wax_; four were contaminated with thiacloprid at 1.9 to 7.8 ppb, contributing 0.08 to 0.31 points to the HQ_wax_. The mean HQ_wax_ score across all samples was 2,155 ± 192.4. The majority of this score came from the presence of varroacides (71.1%), followed by insecticides (28.3%) and fungicides (0.5%), while herbicides contributed minimally (<0.01%). Several MOA groups significantly increased the HQ when multiple products of that same MOA were detected. All wax samples had at least two group 1 acetylcholinesterase inhibiting products with a maximum of seven detected in a single sample. The HQ_wax_ increased significantly when more than two products were detected (χ^2^ = 4.97, df = 1, n = 108, p = 0.026), raising the mean HQ_wax_ from 1,539.6 ± 168.9 to 2,416.4 ± 258.5. All wax samples had at least one product of MOA insecticide group 3 (3.NaCh, sodium channel modulators) with up to 12 different products with this MOA detected in a single sample. In wax, 14.8% samples (n = 16) were free of MOA insecticide group 19 (19.Octo, octopamine receptor agonists) residues and had a mean HQ_wax_ = 1,335.8 ± 174.8, while 33.3% (n = 36) had one group 19 residue HQ_wax_ = 1,940.8 ± 272.3 and the remaining 51.9% had (n = 56) two detected with a HQ_wax_ = 2,545.6 ± 318.8. Seven wax samples were positive for an insecticide residue with an unknown MOA; these few samples had significantly elevated HQ_wax_ scores (χ^2^ = 28.37, df = 1, n = 108, p < 0.0001; MOA group unknown present HQ_wax_ = 5,801.7 ± 1,933.0 vs absent HQ_wax_ = 1,912 ± 133.0;). Fungicide MOA did not influence the total HQ_wax_ score except for MOA fungicide group M (M.Multi, multi-site contact activity) (χ^2^ = 8.39, df = 1, n = 108, p = 0.004). When present, samples with group M fungicides had significantly elevated HQ_wax_ = 2,502.7 ± 267.7 compared to samples where they were absent HQ_wax_ = 1,429.2 ± 135.6.

### Pesticide Prevalence and HQ in Different Operations and Foraging Environments

The participating commercial beekeepers moved their colonies among three different foraging environments: crop pollination, honey production, and holding yards. Only beebread samples were regularly collected and analyzed across all sampling time points in this study. The average total HQ_bbread_ was not significantly different among operations but the number of total pesticide residues in beebread samples varied significantly (Fig. S4, HQ_bbread_: χ^2^ = 4.00, df = 2, n = 147, p = 0.135; Fig. S4, number of residues: χ^2^ = 130.28, df = 2, n = 147, p < 0.0001) as did the total number of “relevant” pesticides χ^2^ = 44.89, df = 2, n = 147, p < 0.0001).

Each participating commercial beekeeper had a different migration route (Fig. 1), and so the differences in pesticide prevalence and abundance among operations is not surprising. Clear peaks in the HQ_bbread_ and number of residues detected occurred when colonies were in or had just been moved out of certain specialty crops, especially citrus, apple, cranberry, and cucumber (Table 1). Notably, in OP1, HQ_bbread_ was significantly higher and the number of pesticides detected greatest when colonies were sampled in May 2007 immediately after apple pollination. In OP2, the HQ_bbread_ was elevated during citrus bloom and late season cucumber pollination. Late cucumber pollination was associated with significantly more pesticide residues than any other crop. In OP3, HQ_bbread_ and pesticide residues were highest when bees were foraging in citrus groves at the start of the study and were also elevated during cranberry pollination (see S1, Fig. S5).

Overall, the total HQ_bbread_ is significantly elevated in pollination environments compared to honey production or holding yards (Fig. S6, top row, χ^2^ = 39.13, df = 2, n = 147, p < 0.0001). Of the beebread samples that had total HQ_bbread_ score greater than 1,000 (15%; n = 22), the majority were collected in March (n = 9) and May (n = 9) during pollination of apple or lowbush blueberry.

When the HQ_bbread_ was subdivided into the four categories of insecticides, fungicides, herbicides, and varroacides, the trends of elevated risks during pollination were consistent across all pesticide groups except for varroacides (Fig. S6). Varroacide levels increased significantly in beebread samples collected while colonies were held in holding yards, presumably because these were the times when beekeepers applied treatments for *Varroa* control.

Fungicide prevalence was low outside of pollination events and was completely absent in 54% and 44% of samples taken from colonies in holding yards and honey production yards, respectively. Only 9% of beebread samples taken from colonies during pollination events were absent of fungicides. These few fungicide-free beebread samples occurred early in the season during citrus and apple pollination. Of the 8.8% of analyzed samples that had a fungicide with an HQ_bbread_ > 50 (HQ_bbread fungicide_ range = 88.3–239.6), all were collected during blueberry (n = 9) and cranberry (n = 4) pollination. Total fungicide residues measured in ppb per sample for in-hive beebread and wax are frequently high (bee bread: mean = 1,706.4 ppb, max = 26,600 ppb; wax: mean = 1,137 ppb, max = 53,704.8 ppb), but were rarely detected in live in-house bees at the start of the season (mean = 1.32 ppb, max = 35.8 ppb).

**Figure 2:**
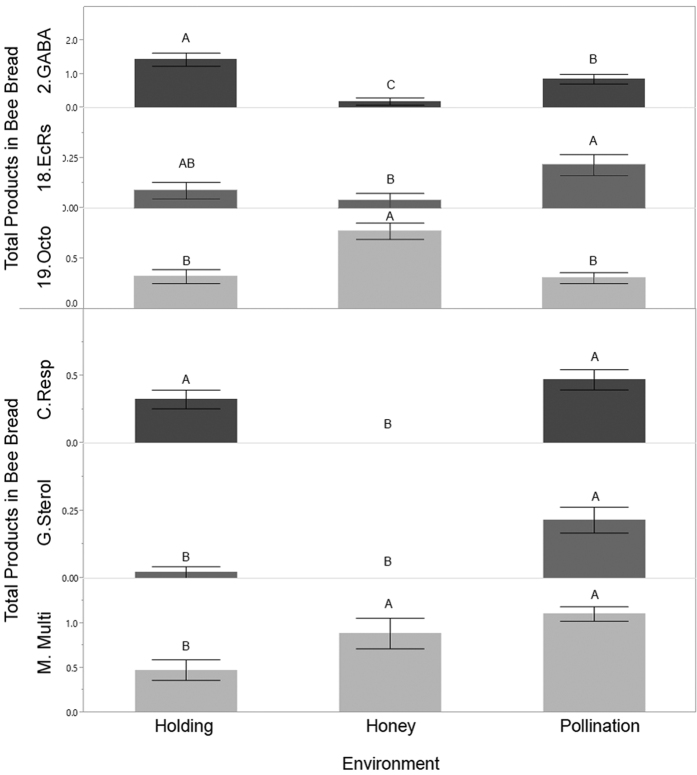
The mean number (+− SE) of products found in bee bread, grouped by their MOA, which differed between environments (as indicated by different letters in each group).

Environment strongly influenced the number of products of a specific MOA found in a beebread sample (Table 2). For insecticides, MOA group 18 (18.EcRs, ecdysone receptor agonists) were more prevalent in pollination environments and reduced when colonies were placed for honey production (Fig. 2), while insecticide MOA group 2 GABA-gated chloride channel blockers were highest in holding yards (predominantly because of endosulfan residues). MOA group 19 octopamine receptor agonists were highest during honey production, which is attributed entirely to DMPF residues, a breakdown product of Amitraz. Several fungicide MOA groups were also elevated during pollination. These included fungicides from the groups C (C.Resp, respiration), G (G.Sterol, sterol biosynthesis in membranes, and M (M.Multi, multi-site contact activity) MOA groups (Fig. 2).

HQ_wax_ at the start of the study did not differ among operations (χ^2^ = 0.16, df = 1, n = 54, p = 0.69), although the total number of relevant pesticides varied between the wax of the two operations sampled (χ^2^ = 11.88, df = 1, n = 54, p = 0.0006), with significantly higher relevant residues in OP3 (mean = 4.22 ± 0.54) compared to OP1, both when the packages in OP1 were included (mean = 2.44 ± 0.17) or excluded (mean = 2.82 ± 0.27).

### Pesticides and colony survivorship during the entire beekeeping season

As previously reported in vanEngelsdorp *et al.*^26^, colony survival varied among operations (Fig. S4) with OP2 experiencing the fewest colony losses. OP2 managed their colonies at the apiary level, equalizing colony strength and replacing dead colonies throughout an apiary and the season, while OP1 and OP3 managed for survival at the individual colony level. For all operations, we considered a colony to be dead when it had no adult bees in the hive at the time of inspection.

**Figure 3:**
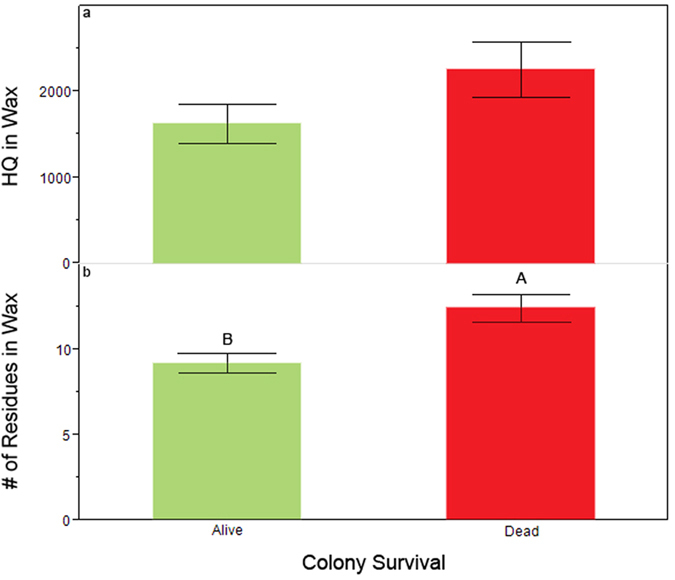
(**a**) Mean HQ (±S.E) and (**b**) mean number of total residues (±S.E) found in wax comb in established colonies that survived the entire beekeeping season (green) compared to those that died (red). Significant differences (α = 0.05) indicated by different letters.

The mean number of residues detected in brood nest wax samples at the start of the study did not differ between colonies that survived versus those that died over the course of the study (χ^2^ = 0.11, df = 1, n = 108, p = 0.736). This analysis included both established colonies and colonies that were installed as packages on drawn comb that had previously had only honey stored in it (as such, colony type strongly influenced the statistical model; χ^2^ = 12.31, df = 1, n = 108, p = 0.0005). Colonies established from packages had significantly fewer total and relevant residues in their wax (6.26 ± 0.55 and 2.11 ± 0.17, respectively) when compared to established colonies (12.0 ± 1.03 and 3.54 ± 0.33, respectively) at the first sampling period (t_52_ = 3.93, p = 0.0002; t_52_ = 3.91, p = 0.0003, respectively). Because of the differences in pesticide residues in packages and established colonies, we excluded packages from the analysis for colony survival. At the start of the study, HQ_wax_ was not significantly different between colonies that lived or died (Fig. 3a, χ^2^ = 1.88, df = 1, n = 70, p = 0.17). However, established colonies that died during the season had significantly more total pesticide residues in their wax over all sampling periods than did colonies that lived (Fig. 3b, χ^2^ = 7.29, df = 1, n = 70, p = 0.0069). Increased exposure followed a similar pattern for relevant pesticides (Died: mean = 3.70 ± 0.27 vs. Survived: 3.0 ± 0.20), but was not significant statistically (χ^2^ = 2.25, df = 1, n = 70, p = 0.13).

**Figure 4:**
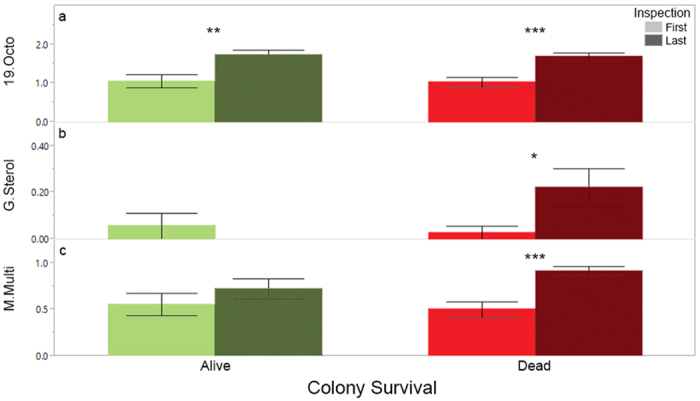
Mean number of total residues detected in wax ± S.E. for different MOA that changed significantly over time during the course of study; (**a**) Insecticide MOA group 19 (octopamine receptor agonists); (**b**) Fungicide MOA group G (sterol biosynthesis in membranes); (**c**) Fungicide MOA group M (multi-site contact activity). Wax samples taken at first inspection light colored, and at last inspection dark colored. Significant differences indicated: *p < 0.05; **p < 0.01; ***p < 0.001.

Additionally, we analyzed the total pesticide residues by grouping them according to their MOA. The number of products that are MOA group 19 insecticides (octopamine receptor antagonists) increased from the first sampling period to the last sampling period, irrespective of whether colonies lived or died (Fig. 4a, χ^2^ = 24.72, df = 1, n = 108, p < 0.0001). This group is exclusively comprised of the breakdown products of the varroacide amitraz, which likely reflects the use of this product for the control of *Varroa* over the course of the season. In colonies that died, the total number of group G fungicides (sterol biosynthesis in membranes) in wax increased between first and last sampling periods (Fig. 4b, Died: χ^2^ = 5.86, df = 1, n = 72, p = 0.0154; Survived: χ^2^ = 1.41, df = 1, n = 36, p = 0.23). A similar increase was seen in fungicides with group M (multi-site contact activity) MOA (Fig. 4c, Died: χ^2^ = 16.36, df = 1, n = 72, p < 0.0001; Survived: χ^2^ = .09, df = 1, n = 36, p = 0.30).

**Figure 5:**
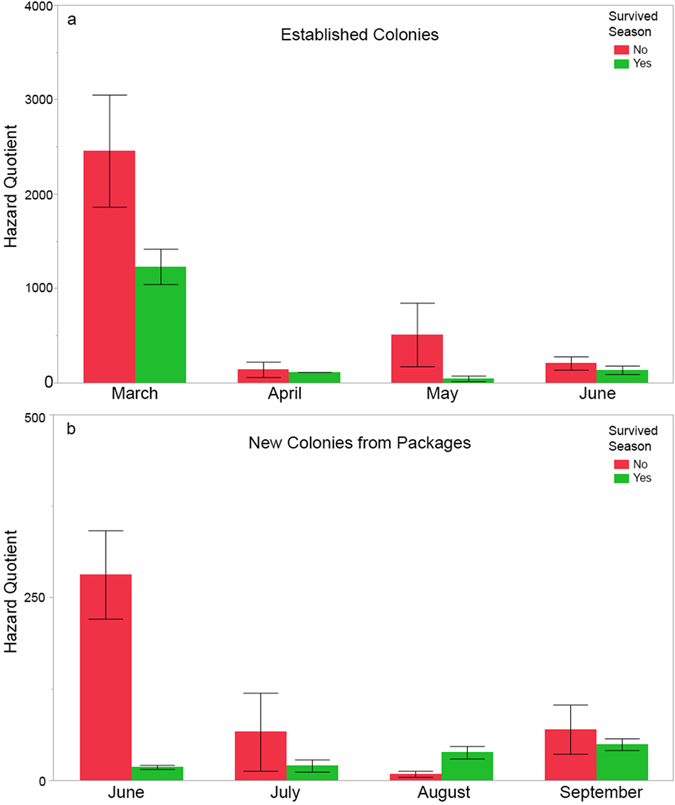
Mean HQ_bbread_ ± SE and colony survival. Mean HQ_bbread_, segregated by colony survival during the beekeeping year. HQ_bbread_ in the first half of the beekeeping season is significantly elevated in established colonies that perish during the beekeeping year. Red = all colonies in the pooled sample die before Jan 2008, Green = all colonies in the pooled sample live. (**a**) established colonies from March–June; (**b**) new colonies established from packages from June-September.

**Figure 6:**
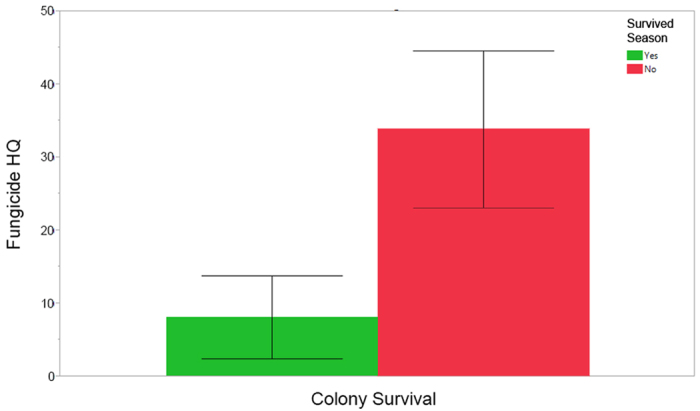
Mean fungicide HQ_bbread_ (±S.E.) during the active beekeeping season from March through September, segregated by colony survival. Red = pooled samples in which all the colonies die before the end of the year; green = pooled samples where all colonies survived the season.

Total HQ_bbread_ was elevated in established colonies during the first half of the beekeeping season from March through June that subsequently died during the beekeeping year (Fig. 5a, HQ_bbread_: χ^2^ = 10.79, df = 1, n = 34, p = 0.0010). The HQ_bbread_ varied significantly by collection date, with the highest scores detected in March (Fig. 5a, Collection Date: χ^2^ = 11.32.32, df = 3, n = 34, p = 0.0101). Insecticides were the greatest contributor to the HQ_bbread_ for these colonies, contributing 940.9 ± 157.6 points to colonies that died during the season compared to 448.5 ± 151.0 points to colonies that survived the entire beekeeping season. We separately analyzed the new colonies (packages) installed on drawn honey comb and determined that an elevated HQ_bbread_ was not associated with colony losses in these newly established packages early in the beekeeping season from March-June (χ^2^ = 0.05, df = 1, n = 15, p = 0.821). However, later in the season (from June through September), packages showed a similar pattern of elevated HQ_bbread_ in colonies that perished (Fig. 5b, χ^2^ = 5.48, df = 1, n = 19, p = 0.0193), suggesting that any advantage to providing colonies with “clean” comb only slightly delayed possible links with pesticide buildup and colony mortality. The HQ_bbread_ scores in packages did not vary by collection date (χ^2^ = 1.93, p = 0.5861). We also examined if colonies that survived differed in their HQ_pesticide category_ compared to colonies that perished, and only fungicides were significant. Since fungicides are only detected during the active beekeeping season, we focused on samples collected between March-September. During this time period, HQ_bbread fung_ was lower in colonies that lived compared to levels in those that perished (Fig. 6, χ^2^ = 5.54, df = 1, n = 82, p = 0.0186). Collection date had no significant influence on the model (χ^2^ = 4.75, df = 6, n = 82, p = 0.5766).

### Pesticides and imminent colony death

**Figure 7:**
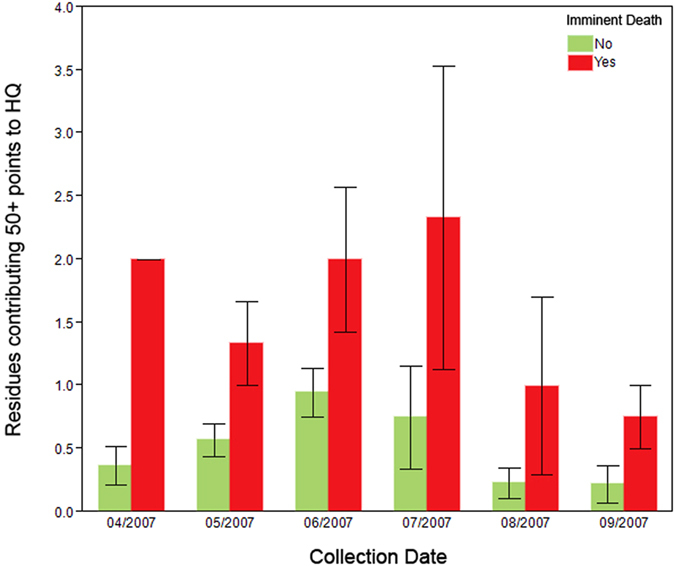
Imminent colony loss and number of residues contributing 50 + to HQ_bbread_. The mean number of residues (±S.E.) excluding miticides used for *Varroa* treatment contributing at least 50 points to the total HQ_bbread_, segregated by imminent death. Red = pooled samples in which at least one colony dies before the next sampling period; green = pooled samples where all colonies are still alive the next inspection period.

An elevated HQ_bbread_ was not predictive of imminent (within ~30 days) colony loss (χ^2^ = 2.15, df = 1, n = 107, p = 0.14). In contrast, the total number of products found in beebread trended toward being elevated in colonies that perished (χ^2^ = 3.59, df = 1, n = 107, p = 0.0582), while the number of relevant products contributing at least 50 points to the total HQ_bbread_ was significantly elevated in colonies that died before the next sampling period during the beekeeping season from April through September (Fig. 7, χ^2^ = 14.06, df = 1, n = 107, p = 0.0002). Imminent colony death also varied by collection date (Fig. 7, χ^2^ = 18.34, df = 5, n = 107, p = 0.0025), but there was no significant interaction of collection date and HQ_bbread_ (χ^2^ = 6.62, df = 5, n = 107, p = 0.25).

**Figure 8:**
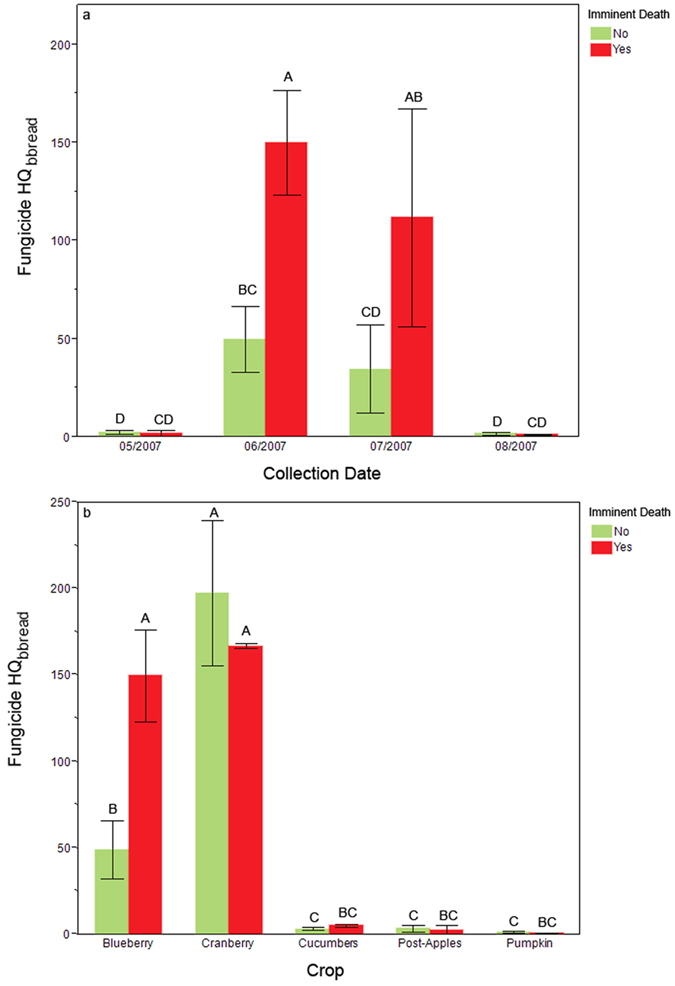
Fungicide contributions to HQ_bbread_ by sampling period and crop pollinated. During the summer season elevated mean fungicide HQ_bbread_ (±S.E.) were linked with colony death 1 month later in June and July. Fungicide HQ varied significantly by crop. (**a**) Fungicide HQ by sampling period; (**b**) Fungicide HQ by crop. Different letters indicate significant differences. Red = pooled samples in which at least one colony dies before the next sampling period; green = pooled samples where all colonies are still alive during the next inspection.

Despite their low overall contribution to the HQ_bbread_, fungicides were significantly elevated during the summer season from May-August in colonies that died within ~30 days of sampling (Fig. 8a, χ^2^ = 5.72, df = 1, n = 78, p = 0.0168). The fungicide HQ_bbread_ varied significantly by collection date during that same period (χ^2^ = 46.50, df = 3, n = 78, p < 0.0001). No fungicides were detected during citrus pollination. Samples from citrus were thus excluded when determining how HQ_bbread Fung_ varied by crop. HQ_bbread Fung_ was elevated during blueberry and cranberry pollination compared to other crops (Fig. 8b, Crop: χ^2^ = 138.90, df = 4, n = 55, p < 0.0001), but only elevated fungicide scores associated with blueberry pollination were linked with imminent colony loss (t_20_ = 2.29, p = 0.033).

To determine if the MOA of particular pesticides was implicated in imminent colony loss, we examined if the total number of products with the same MOA was elevated in beebread in colonies that perished before the next sampling period. No particular mode of action was significantly associated with imminent colony loss, though there was a trend toward elevated residues of insecticide MOA group 18 insecticides (ecdysone receptor agonists) in colonies that died (mean = 0.25 ± 0.08) compared to colonies that lived (mean = 0.10 ± 0.03) (χ^2^ = 3.44, df = 1, n = 139, p = 0.063). A similar pattern was seen with insecticides categorized as an unknown MOA (χ^2^ = 2.94, df = 1, n = 139, p = 0.087), which were somewhat elevated in colonies that died (mean = 0.14 ± 0.07) compared to those that lived (mean = 0.05 ± 0.02) to the next sampling period.

### Specific pesticides and colony mortality

**Figure 9:**
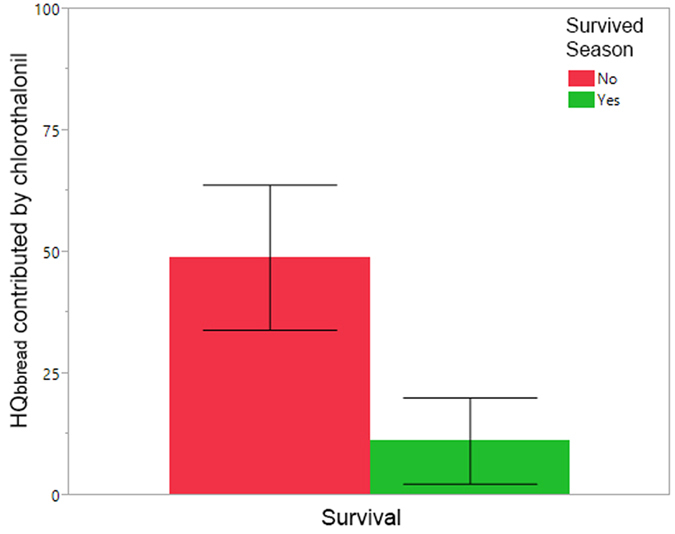
Mean HQ_bbread_ (±S.E.) contributed by chlorothalonil in colonies that perished during the beekeeping season in bee bread samples collected May-August, months when this fungicide was detected.

The insecticide fipronil was found in one adult bee sample (see above), in one beebread sample, and in one wax sample. These samples came from different colonies, and in all cases the colony from which the sample was collected died before the next sampling event. The presence of the fungicide chlorothalonil in pooled beebread samples was common and often exorbitant. Of the 147 beebread samples analyzed, 87 had detectable levels of chlorothalonil, with 19.5% (n = 17) contaminated at more than 1,000 ppb and 13.8% (n = 12) at more than 10,000 ppb. In beebread, the maximum chlorothalonil residue was 26,600 ppb, eight times higher than the maximum varroacide residue detected (3,260 ppb for coumaphos). Colonies that perished during the beekeeping year had significantly higher HQ_bbread chlorothalonil_ during the summer season from May-August than colonies that survived (Fig. 9, χ^2^ = 5.62, df = 1, n = 54, p = 0.0177).

### Pesticide prevalence, load and queen replacement events

**Figure 10:**
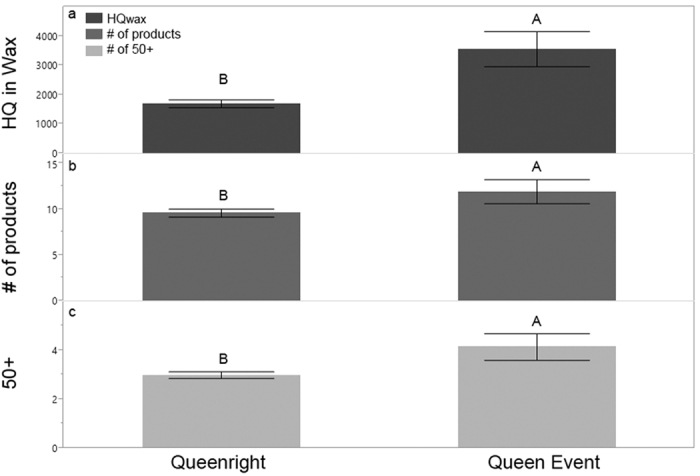
Queen events and pesticide contamination in wax. (**a**) Mean HQ_wax_ (±S.E.) for colonies that experienced a queen event compared to colonies that did not lose or replace their queen (queenright), (**b**) mean total number of pesticide residues detected, (**c**) mean number of relevant pesticide residues (50+) detected. Significant differences (α = 0.05) indicated by different letters.

**Figure 11:**
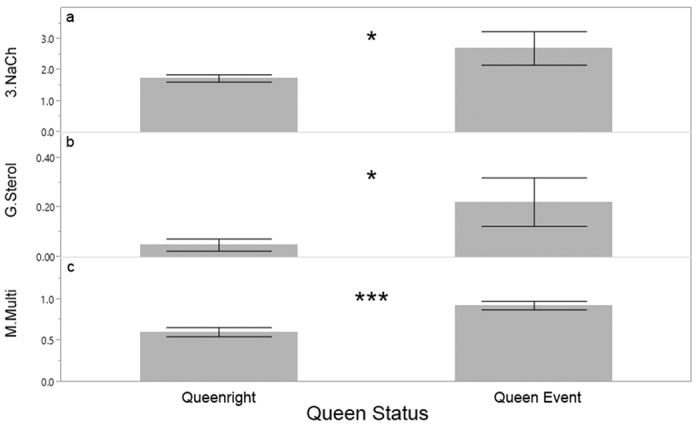
Mean number of products with a given MOA (±S.E.) detected in wax from colonies that never experience a queen event (queenright) compared to number of products in wax from colonies that experienced a queen event (right). Colonies that experienced queen events had more (**a**) Insecticide group 3 (sodium channel modulators) products, (**b**) Fungicide group G (sterol biosynthesis in membranes) and (**c**) Fungicide group M (multi-site contact activity) products. Difference are indicated (*p < 0.05, ***p < 0.001).

As described in vanEngelsdorp *et al.*^26^, one of the leading predictors of imminent colony mortality was a queen event—that is, evidence that the queen was recently replaced (e.g., presence of a virgin queen), was being replaced (e.g., supersedure cells), or the colony was queenless. Colonies that were diagnosed with this condition were more than three times as likely to die over the next ~50 days. All three models (HQ_wax_, total pesticide residues, and relevant residues) were higher in the wax of colonies that experienced queen events (Fig. 10: HQ_wax_: χ^2^ = 22.38, df = 1, n = 108, p < 0.0001; Total products: χ^2^ = 5.04, df = 1, n = 108, p = 0.025; relevant pesticides: χ^2^ = 8.08, df = 1, n = 108, p = 0.005). All colonies with HQ_wax_ scores above 6,500 (n = 5) experienced queen events during the season. When analyzed by MOA, the number of group 3 insecticides (sodium channel modulators) was significantly higher in the wax of colonies that experienced a queen event than colonies that remained queenright (Table 3, Fig. 11). Though their presence was much lower, a similar pattern was seen with group G fungicides (sterol biosynthesis in membranes) and group M fungicides (multi-site contact activity) (Table 3, Fig. 11).

## Discussion

The exposome is a measure of all exposures within an individual’s lifetime. The term was first used by cancer epidemiologist C. Wild to help elucidate the role environmental exposures have on human health^46^. Here we apply this concept for the first time to the superorganism of a honey bee colony, which enables us to document part of the pesticide exposome in colonies pollinating commercial crops in the northeastern United States. In doing so, we tracked pesticide levels in wax and beebread over time and summarized these levels using three different approaches (total number of product residues, total number of relevant product residues, and calculating a HQ score) then linked them to colony mortality (seasonal or imminent) and queen events. We found that pesticide contamination in stored beebread and wax comb was associated with colony mortality and increased queen replacement.

The three models implemented are imperfect estimates of pesticide burden, as they neither consider synergistic or antagonistic interactions among products nor the ability of bees to detoxify products. The calculations also only included the 171 products we tested and that occurred at levels above the limit of detection (Table S1), and does not account for adjuvants^47^,^48^ and other products not included in the screening (such as glyphosate, which requires a separate, costly analysis). Further, our first model—the simple sum of detected products—completely ignores the central tenant of toxicology: the dose makes the poison^49^,^50^. As such, we attempted to exclude possibly insignificant trace residues with our second model that only counts the number of relevant product detections contributing at least 50 points to the HQ score. Despite the significant limitations of the three models employed here, we find clear relationships between colony health and the total and relevant number of product residues and the HQ scores in wax, beebread, or both.

The total number of products found in wax was higher in colonies that died compared to those that did not (Fig. 3), while increased HQ scores in wax were tied to an increased rate of queen replacement (Fig. 10). Beebread samples taken from colonies that died less than 30 days after sample collection had more relevant products (50+) compared to colonies that survived (Fig. 7). Beebread samples taken during the active beekeeping season had higher HQ_bbread_ scores when they originated from colonies that subsequently died by the end of the study compared to samples taken from their surviving counterparts (Fig. 5).

The ability of our models to identify potential risk associated with multiple pesticide exposures is also evident when one considers how few individual products were directly linked with colony mortality. The rarely occurring fipronil was only detected in three different colonies, and in all cases those colonies died by the next sampling period. The very commonly detected fungicide chlorothalonil occurred at much greater concentrations in beebread samples taken from colonies that died during the beekeeping season (Fig. 9). Using the numbers of residues and HQ models to summarize part of the colony’s exposome, we were able to link broad pesticide categories (e.g., fungicides; Figs 6 and 8) to colony mortality. Further, these models enabled us to highlight the potential role of certain MOAs with colony health outcomes. Specifically, we found that the number of group G and group M fungicides (the latter includes the aforementioned chlorothalonil) increased significantly in the wax of colonies that died over the course of study. They were also found more numerously in the wax of colonies that experienced a queen event (Fig. 11). We also found that the presence of insecticide MOA group 18 tended (p = 0.058) to be higher in colonies that would die than those that would not. The latter is particularly concerning as this group is classified as relatively non-toxic to bees, and as such the current regulatory authority does not prevent or limit their use during bloom when bees are actively foraging.

Despite the large—and admittedly overly simplistic—assumptions that underlie our approach to summarizing the pesticide exposome in commercial beekeeping operations, our models were able to highlight relationships between multiple real world pesticides residues and increased colony mortality and queen longevity. These associations would have been missed had we simply looked for links between the presence or absence of each specific product and different colony outcomes (e.g., Odds ratios^51^). Additionally, these models were able to easily differentiate operations and landscapes with different levels of exposure “risk”. Honey bees have long been identified as environmental bioindicators^52^,^53^. In our study, we documented a wide range in HQ scores and number of residues in beebread samples over time. We found significant differences in pesticide exposures among different operations and the environments in which the colonies were foraging (Figs S4, S5 and S6, and Table 2). All categories of pesticides, except varroacides, were elevated in HQ_bbread_ collected during pollination events (Fig. S6) when compared to honey producing or holding yard environments, and this increase was associated with increased colony mortality (Figs 5 and 7).

Our findings highlight specific environments that may be particularly risky for pollinators, which may lead to recommendations to mitigate colony mortality. Pollination environments almost invariably contribute more pesticide residues, potentially because of drift onto nearby non-cultivated plants that present a season-long route of pesticide exposure for honey bees^54^. For instance, as previously mentioned, we found high numbers of group 18 insecticides in beebread associated with increased mortality during the active beekeeping season (Fig. 7). We also found that fungicides made a greater contribution to HQ_bbread_ scores in colonies that died over the active bee season (Fig. 6). The average number of products found in beebread taken from colonies pollinating cranberry was 12.5, of which 4 were group 1 insecticides and 1 was a group 18 insecticide. The cranberry pollination environment also resulted in a relatively high HQ_Fung_ (Fig. 8b). Thus, we would predict that pollination of cranberry would result in elevated mortality. Longitudinal monitoring of colonies is expensive and difficult, and our sample size of 16 colonies pollinating cranberry is not sufficient to make valid direct linkages between cranberry pollination and increased mortality. Nonetheless, our in-hive pesticide exposome summation models suggest cranberry is a high-risk crop for honey bees (or at least was in 2007). A better understanding of these risks may help further elucidate mitigation practices; which, if implemented, would reduce risks associated with exposure. For instance, if a beekeeper is pollinating a crop that has a history of MOA group 1 insecticide use, they should consider avoiding varroacides with this same mode of action (e.g., coumaphos). Beekeepers could also request producers avoid certain groups of insecticides (e.g., those belonging to insecticide MOA group 18), which are currently considered ‘bee safe’ as they have little effect on adult bees, but their presence is correlated with increased colony mortality (Fig. 7). There is a growing body of evidence of the harmful impacts of fungicides on pollinator health^55^,^56^,^57^. Our correlative studies suggest that group G and group M fungicides are associated with poor colony health. If confirmed by future experiments, beekeepers could request that producers apply fungicides with different modes of action as a condition in their pollination contracts.

The high risk of exposure while pollinating (Fig. S6) suggests that beekeepers may wish to alternate among environments to provide colonies sufficient time to recuperate from high risk environments^21^. We found a wide range of HQ scores in our samples collected in 2007, with scores above our 1,000 threshold found in a variety of crops (Table 1). Caution, however, is needed when inferring that these results are relevant in the present day. This study was conducted in 2007, and the crop-protection products used on different crops has almost certainly evolved in the interim. Nonetheless, the high diversity of products found in different pollination environments suggests that more research is needed to understand how these products, many of which would not have been sprayed while bees were foraging, persist in the environment and become sequestered in honey bee colonies, with recent research suggesting the residues persist via uncultivated crops potentially exposed to pesticide drift^58^.

As repeatedly mentioned, the models used here to summarize the complex number of interactions occurring within the colony environment are additive, and thus may underestimate the real threat posed to honey bee colonies, as many products interact synergistically. Toxicologists and other scientists have called for the development of more robust models to better estimate pesticide risk to pollinators^29^,^59^,^60^. Use of models, even imperfect ones as in this study, may help to highlight such associations, which can then be verified through hypothesis-driven experimentation. Applying these rudimentary models to real-world data facilitates the development of much-needed recommendations to mitigate problems associated with pesticide exposure, thus helping to reduce pollinator exposure risk, which benefits both beekeepers and the producers who rely on honey bees for pollination.

## Additional Information

**How to cite this article**: Traynor, K. S. *et al.* In-hive Pesticide Exposome: Assessing risks to migratory honey bees from in-hive pesticide contamination in the Eastern United States. *Sci. Rep.* **6**, 33207; doi: 10.1038/srep33207 (2016).

## Supplementary Material

Supplementary Information

## Figures and Tables

**Table 1:** Mean Bee Bread Hazard Quotients ± SE and Mean Number of Pesticide Residues ± SE by Operation and Crop Exposure.

Date	Operation 1				Operation 2					Operation 3		
	Crop	HQ_bbread_	Residues	50+	Crop	HQ_bbread_	Residues	50+	Crop	HQ_bbread_	Residues	50+
03/2007					Citrus	1102.7 ± 192.0 a	7.71 ± 0.18 cd	2.57 ± 0.20 a	Citrus	2240.6 ± 393.6 a	10.6 ± 0.93	2.8 ± 0.37 ab
04/2007	Holding	70.8 ± 23.4 b	2.92 ± 0.43 c	0.5 ± 0.19 bcd								
05/2007	Post apples	1735.8 ± 304.5 a	6.83 ± 0.34 a	1.08 ± 0.08 a	Blueberry	19.9 ± 5.2 c	6.57 ± 0.53 d	0 d	Holding	88.2 ± 27.0 c	8.0 ± 1.05	0.6 ± 0.4 c
06/2007	Blueberry	147.9 ± 34.5 b	5.27 ± 0.27 b	0.91 ± 0.25 ab	Cucumber	222.9 ± 53.8 bc	10.0 ± 0.53 bc	0.71 ± 0.18 cd	Blueberry	336.8 ± 100.1 bc	10.5 ± 0.29	2.25 ± 0.48 b
07/2007	Honey	36.1 ± 14.2 b	4.82 ± 0.30 b	0.18 ± 0.18 d					Cranberry	938.0 ± 220.5 b	12.5 ± 0.87	3.5 ± 0.29 a
08/2007	Pumpkin	33.8 ± 11.4 b	3.60 ± 0.27 c	0.2 ± 0.13 cd	Holding	455.4 ± 172.5 abc	11.1 ± 0.94 b	1.71 ± 0.42 b				
09/2007	Honey	70.7 ± 11.1 b	4.70 ± 0.21 b	0.3 ± 0.15 cd	Cucumber	733.0 ± 516.4 ab	15.3 ± 1.43 a	1.71 ± 0.29 b				
10/2007									Honey	76.5 ± 27.9 c	6.5 ± 0.87	0.25 ± 0.25 c
11/2007	Holding	103.8 ± 22.3 a	4.88 ± 0.44 b	0.75 ± 0.25 abc	Holding	468.7 ± 173.4 abc	9.0 ± 1.07 bcd	2.0 ± 0.44 ab	Honey	161.5 ± 2.5 bc	11.0 ± 1.00	1.5 ± 0.5 bc
01/2008					Holding	211.7 ± 102.2 bc	8.6 ± 1.29 bcd	1.29 ± 0.18 bc				

OP1 HQ_bbead_: χ^2^ = 350.70, df = 6, n = 74, p < 0.0001, Residues: χ^2^ = 23.19, df = 6, n = 74, p = 0.0007.

OP2 HQ_bbread_: χ^2^ = 21.99, df = 6, n = 49, p = 0.0012, Residues: χ^2^ = 32.97, df = 6, n = 49, p < 0.0001.

OP3 HQ_bbread_: χ^2^ = 142.68, df = 5, n = 24, p < 0.0001, Residues: χ^2^ = 10.32, df = 5, n = 24, p = 0.0665.

Significant differences (α = 0.05) in HQ_bbread_ and number of residues detected within a single operation (column) indicated by different letters.

**Table 2:** The number of residues detected in bee bread collected from colonies in different beekeeping environments, grouped by MOA.

	MOA^a^	Number of Residues Detected in bee bread			χ^2^	p
		Holding	Honey	Pollination		
Insect & Varroa	1.AChE	1.48 ± 0.12	1.59 ± 0.17	1.72 ± 0.11	2.00	0.37
	2.GABA	1.43 ± 0.20	0.19 ± 0.11	0.85 ± 0.14	22.15	<0.0001*
	3.NaCh	1.58 ± 0.15	1.19 ± 0.09	1.65 ± 0.12	3.02	0.22
	18.EcRs	0.09 ± 0.04	0.04 ± 0.04	0.22 ± 0.05	6.59	0.037*
	19.Octo	0.32 ± 0.07	0.78 ± 0.08	0.31 ± 0.05	9.79	0.008*
	UN	0.13 ± 0.05	0.04 ± 0.04	0.05 ± 0.03	2.66	0.26
Fung	B.Cyto	0.09 ± 0.04	0	0.04 ± 0.02	3.85	0.15
	C.Resp	0.33 ± 0.07	0	0.47 ± 0.08	21.81	<0.0001*
	G.Sterol	0.01 ± 0.01	0	0.29 ± 0.10	16.68	0.0002*
	M.Multi	0.48 ± 0.12	0.89 ± 0.17	1.11 ± 0.08	14.16	0.0008 *

Differences between the columns occur when the p value from the (Chi square test (χ^2^)) were less than 0.05, as indicated by*.

Mode of Action (MOA): For insecticides and varroacides source: http://www.irac-online.org/modes-of-action/; 1.AChE–Acetylcholineasterase inhibitors; 2.GABA-GABA-gated chloride channel blockers, 3.NaCh–Sodium channel modulators, 18.EcRs-Ecdysone receptor agonists, 19.Octo-Octopamine receptor agonists, UN- Unknown or uncertain MOA; For fungicides source: http://www.frac.info/docs/default-source/publications/frac-code-list/frac-code-list-2016.pdf?sfvrsn=2; B.Cyto-Cytoskeleton and motor proteins; C.Resp–Respiration, G.Sterol-Sterol biosynthesis in membranes, M.Multi-Multi-site contact activity.

**Table 3:** Queen Events and total number of pesticides products found in comb wax at the start of the study, grouped by MOA.

	MOA^a^	Total number of residues in comb wax		χ^2^	p
		Queen Event	Queenright		
Insect & Varroacides	1.AChE	2.29 ± 0.29	2.17 ± 0.12	0.22	0.64
	2.GABA	1.24 ± 0.25	1.28 ± 0.14	0.03	0.86
	3.NaCh	2.71 ± 0.66	1.91 ± 0.14	5.75	0.0165*
	4.nAChR	0	0.06 ± 0.03	1.71	0.19
	7.JH	0.06 ± 0.06	0.02 ± 0.02	0.64	0.43
	18.EcRs	0.41 ± 0.15	0.68 ± 0.10	1.88	0.17
	19.Octo	1.53 ± 0.15	1.34 ± 0.10	0.92	0.34
	UN	0.12 ± 0.08	0.08 ± 0.04	0.27	0.60
Fungicides	B.Cyto	0.06 ± 0.06	0.06 ± 0.03	0.00	0.97
	C.Resp	0.53 ± 0.12	0.32 ± 0.08	1.66	0.20
	D.AAsyn	0.06 ± 0.06	0.04 ± 0.03	0.13	0.72
	E.Sig	0.06 ± 0.06	0.02 ± 0.02	0.64	0.43
	F.LSMI	0.06 ± 0.06	0	2.88	0.09
	G.Sterol	0.24 ± 0.14	0.06 ± 0.03	5.07	0.0244*
	M.Multi	0.88 ± 0.08	0.60 ± 0.07	11.59	0.0007*

Differences between columns are indicated (*) by p values less than 0.05.

Mode of Action (MOA): For insecticides and varroacides source: http://www.irac-online.org/modes-of-action/; 1.AChE–Acetylcholineasterase inhibitors; 2.GABA-GABA-gated chloride channel blockers, 3.NaCh–Sodium channel modulators, 4.nAChR -Nicotinic acetylcholine receptor competitive modulators, 7.JH–Juvenile hormone mimics, 18.EcRs-Ecdysone receptor agonists, 19.Octo-Octopamine receptor agonists, UN- Unknown or uncertain MOA; For fungicides source: http://www.frac.info/docs/default-source/publications/frac-code-list/frac-code-list-2016.pdf?sfvrsn=2; B.Cyto-Cytoskeleton and motor proteins; C.Resp–Respiration, D.AAsyn-Amino acids and protein synthesis, E.Sig-Signal transduction, G.Sterol-Sterol biosynthesis in membranes, F.LSMI-Lipid synthesis and membrane integrity, M.Multi-Multi-site contact activity.
