# Supplementary material for: In-hive Pesticide Exposome: Assessing risks to migratory honey bees from in-hive pesticide contamination in the Eastern United States
Source: Sci Rep. 2016 Sep 15;6:33207. doi: 10.1038/srep33207 (PMC5024099; doi:10.1038/srep33207)
Supplement: Supplementary Information [file srep33207-s1.pdf]

In-hive Pesticide Exposome: Assessing risks to migratory honey bees from in-hive pesticide contamination in the Eastern United States

Kirsten S. Traynor[a], Jeffery S. Pettis[b], David R. Tarpy[c], Christopher A. Mullin[d], James L. Frazier[d], Maryann Frazier[d], Dennis vanEngelsdorp[a*]

Supplemental Information

Supplemental text

S1. Pesticide results in individual operations

OP1: Insecticide levels varied significantly during the sampling period (Fig. S4a, $F_{6,67} = 25.81$, $p < 0.0001$) and had a significant peak in May after apple pollination contributing a mean $HQ_{Insecticide} = 1{,}716.2 \pm 124.3$, while no insecticide residues were detected during pumpkin pollination in August. Varroacide residues occurred throughout the season with no significant differences among sampling periods. Herbicide residues remained consistently low throughout sampling periods. Fungicides varied significantly during the sampling period ($F_{6,67} = 10.65$, $p < 0.0001$), with a significant peak occurring during blueberry pollination ($HQ_{Fung} = 86.5 \pm 9.84$) compared to all other sampling periods where fungicide contamination remained relatively low ($HQ_{Fung} < 4 \pm 11.5$).

OP2: Insecticide contamination varied throughout the beekeeping season with a significant peak during citrus pollination (Fig. S4b, $F_{6,42} = 2.67$, $p < 0.05$). Varroacide residues peaked in August, presumably after *Varroa* treatments and remained elevated throughout the rest of the beekeeping season compared to early sampling periods ($F_{6,42} = 5.68$, $p < 0.0005$). Herbicides, though low contributors to the HQ throughout the collection period, varied significantly ($F_{6,42} = 2.43$, $p < 0.05$) with a peak during citrus pollination and November honey production. Fungicide levels remained low throughout the sampling periods ($F_{6,42} = 5.91$, $p < 0.0005$) with small peaks in June and September during cucumber pollination, significantly elevated above fungicide residues during citrus pollination in March and holding times at the end of the beekeeping year.

OP3: All four categories of pesticides varied significantly throughout the sampling period. The exposure risk of insecticides during citrus pollination was great, with residues contributing 2,204 ± 203 to the HQ and significantly elevated above all other sampling times (Fig. S4c, $F_{5,18} = 15.76$, $p < 0.0001$). Varroacides were also significantly elevated in March during citrus pollination ($F_{5,18} = 18.51$, p <

0.0001), presumably because of early spring *Varroa* treatments. As seen in Op2, herbicides (though low contributors to the HQ throughout the collection period) varied significantly ($F_{5,18} = 8.94$, $p = 0.0002$) with a peak during citrus and cranberry pollination. Fungicides ($F_{6,18} = 22.46$, $p < 0.0001$) peaked in June and July during blueberry and cranberry pollination, contributing 106 ± 16.5 and 182 ± 16.5 points respectively to the HQ.

Supplemental Figures

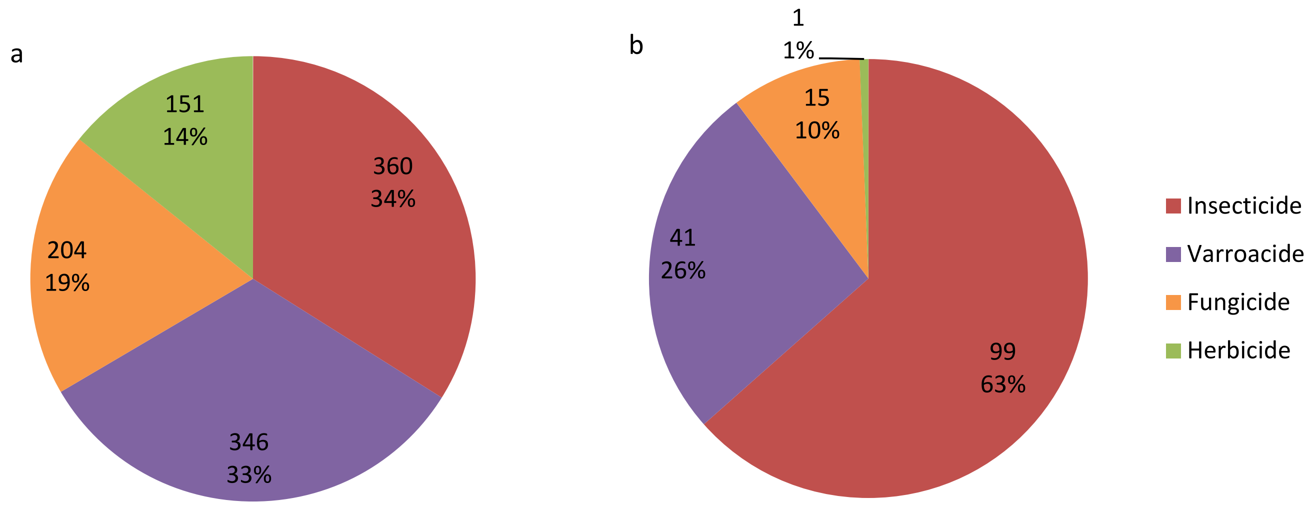

Figure S1. Total residues (a) and relevant residues 50+ (b) detected in bee bread samples, segregated by pesticide category. Insecticides and varroacides each make up a third of all total detects (left), while insecticides make up the majority of the relevant detects. a) Total residue detects = 1,061; b) relevant residue detects = 156. Numerical labels for each pie wedge indicate n (above) and % of total (below).

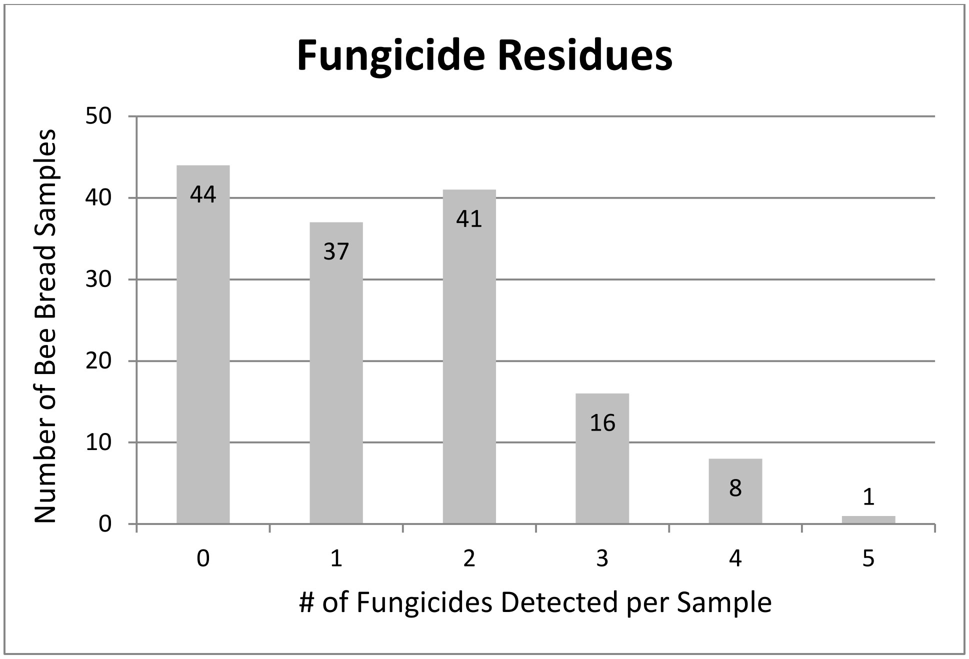

Figure S2. The number of fungicide residues detected per bee bread sample collected over the course of the season.

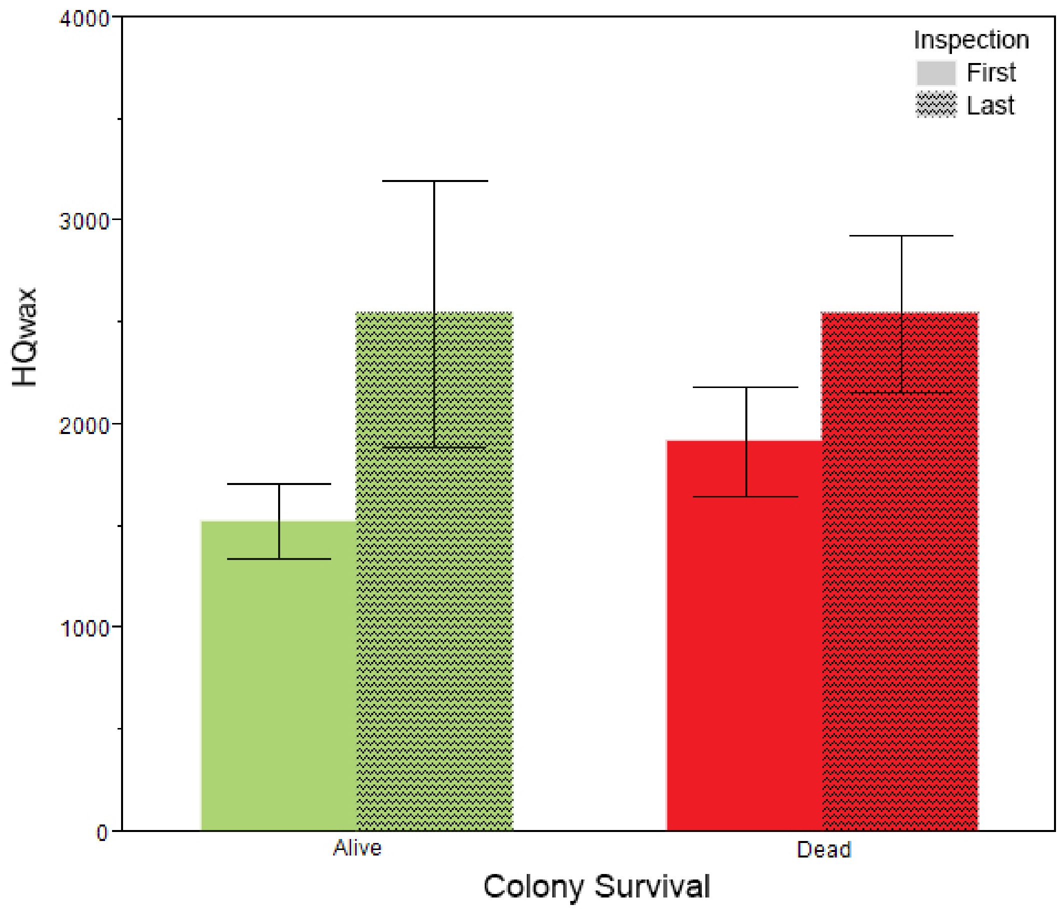

Figure S3. Mean HQ_{wax} (± S.E.) for colonies at the first (solid) and last (pattern) inspection periods for colonies that were alive (green) throughout or died (red) during the beekeeping season.

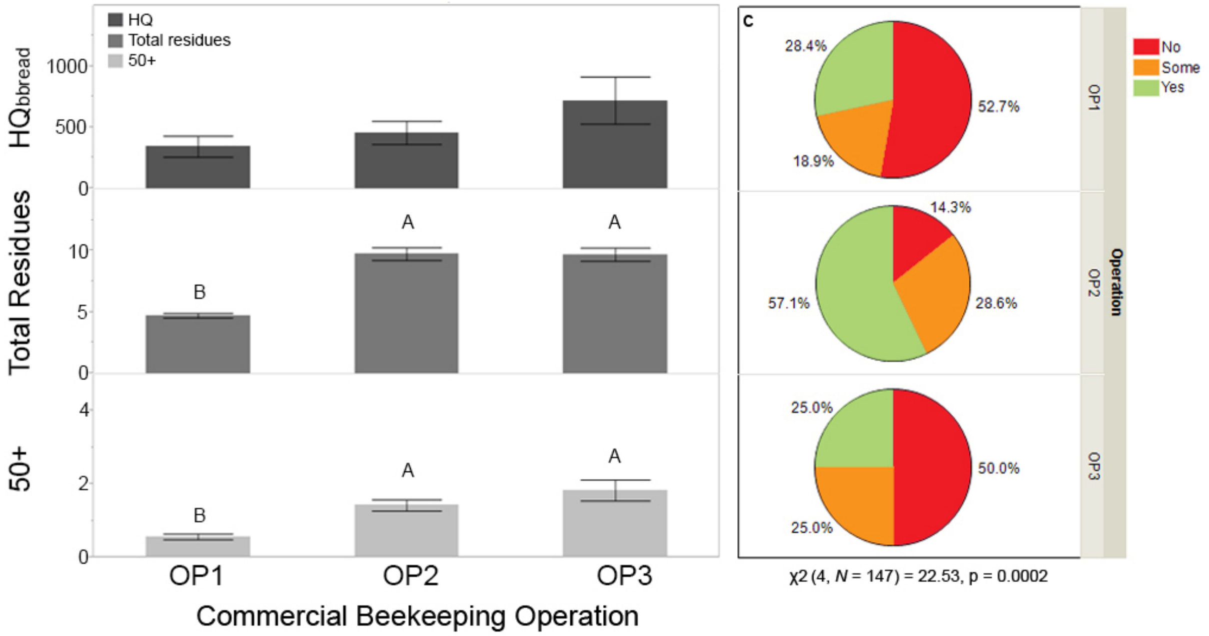

Figure S4. HQ, Pesticide Residues, Relevant Residues (50+) and Colony Survival by Beekeeping Operation
The mean number of pesticide products and the mean number of relevant products contributing 50+ points to the HQ_{bbread} differed between beekeeping operations. Colony survival throughout the beekeeping season also differed between operations; red = all colonies in sampling pool died; orange = mixed pools, where some colonies in the sampling pool died; green = all colonies in the sampling pool survived through Jan 2008.

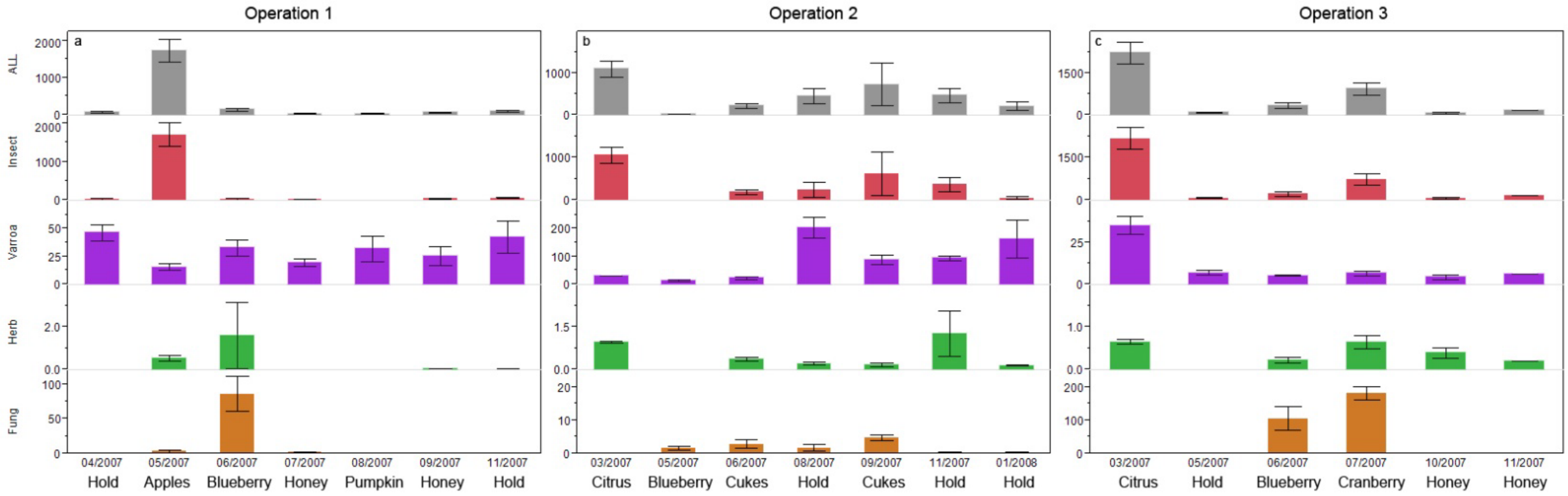

Figure S5. Proportion of HQ score in bee bread samples collected over the season grouped by pesticide categories and segregated by commercial operation. Mean HQ_{bbread} (± S.E.) for all pesticide residues (gray) and divided into the four pesticide categories of insecticides (red), varroacides (purple), herbicides (green) and fungicides (orange) by operation; a = Operation 1, b = Operation 2, c = Operation 3.

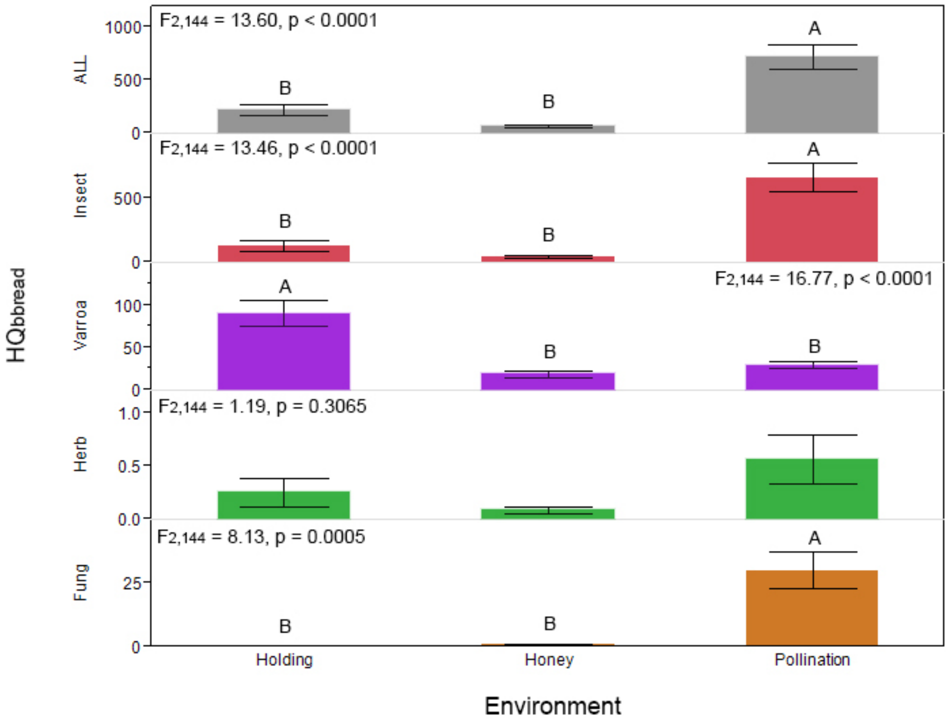

Figure S6. The HQ_{bbread} of samples collected in different colony environments, total pesticide HQ scores and scores broken down by pesticide classification are presented. Mean HQ_{bbread} (± S.E.) for all pesticide residues (gray) and divided into the four pesticide categories of insecticides (red), varroacides (purple), herbicides (green) and fungicides (orange) varied by beekeeping environment with the highest HQ_{bbread} scores during pollination events compared to honey production or holding environments for all components except varroacides. Significant differences indicated by different letters.

Supplemental Tables

Table S1. Pesticide category, mode of action, average LD_{50} and toxicity rank.

Pesticide	Category	Mode of Action[a]	Average LD_{50} (µg/bee)[b]	Toxicity Rank
1-Naphthol (Carbaryl degradate)	Insecticide	1.AChE	0.442	High
3-Hydroxycarbofuran (degradate)	Insecticide	1.AChE	0.115	High
Aldicarb sulfone	Insecticide	1.AChE	83	Low
Aldicarb sulfoxide	Insecticide	1.AChE	1.84	Moderate
Allethrin	Insecticide	3.NaCh	6.1	Moderate
Atrazine	Herbicide	C1.PPII	98.5	Low
Azinphos-methyl	Insecticide	1.AChE	0.179	High
Azoxystrobin	Fungicide	C.Resp	112	Non-toxic
Bifenthrin	Insecticide	3.NaCh	0.0412	High
Boscalid	Fungicide	C.Resp	155	Non-toxic
Captan	Fungicide	M.Multi	135	Non-toxic
Carbaryl	Insecticide	1.AChE	0.442	High
Carbendazim (MBC)	Fungicide	B.Cyto	50	Low
Carfentrazone-ethyl	Herbicide	E.PPO	85.4	Low
Chlorfenapyr	Insecticide	UN	0.12	High
Chlorfenvinphos	Insecticide	1.AChE	2.32	Moderate
Chlorferone (Coumaphos degradate)	Varroacide	1.AChE	5.93	Moderate
Chlorothalonil	Fungicide	M.Multi	111	Non-toxic
Chlorpyrifos	Insecticide	1.AChE	0.0762	High
Coumaphos	Varroacide	1.AChE	5.93	Moderate
Coumaphos oxon	Varroacide	1.AChE	5.93	Moderate
Cyfluthrin	Insecticide	3.NaCh	0.0279	High
Cyhalothrin	Insecticide	3.NaCh	0.183	High
Cypermethrin	Insecticide	3.NaCh	0.188	High
Cyprodinil	Fungicide	D.AAsyn	332	Non-toxic
DDD (DDT degradate)	Insecticide	3.NaCh	6.4	Moderate
DDE (DDT degradate)	Insecticide	3.NaCh	6.4	Moderate
DDT	Insecticide	3.NaCh	6.4	Moderate
Deltamethrin	Insecticide	3.NaCh	0.0434	High
Diazinon	Insecticide	1.AChE	0.169	High
Dicofol	Insecticide	UN	18.6	Low
Dieldrin	Insecticide	UN	0.227	High
Dimethomorph	Fungicide	F.LSMI	30.8	Low
Diphenamid	Herbicide	K3.ICD	2430	Non-toxic
DMA (2,4-Dimethylaniline, Amitraz degradate)	Varroacide	19.Octo	75	Low
DMPF (2,4-Dimethylphenyl	Varroacide	19.Octo	75	Low

formamide, Amitraz degradate)				
Endosulfan 1	Insecticide	2.GABA	7.05	Moderate
Endosulfan 2	Insecticide	2.GABA	7.05	Moderate
Endosulfan sulfate (degradate)	Insecticide	2.GABA	21.8	Moderate
Esfenvalerate	Insecticide	3.NaCh	0.162	High
Ethion	Insecticide	1.AChE	10.7	Moderate
Ethofumesate	Herbicide	N.ILS	50	Low
Famoxadone	Fungicide	C.Resp	25	Low
Fenamidone	Fungicide	C.Resp	93.6	Low
Fenbuconazole	Fungicide	G.Sterol	149	Non-toxic
Fenhexamid	Fungicide	G.Sterol	159	Non-toxic
Fenoxaprop-P-ethyl	Herbicide	A.ACCase	220	Non-toxic
Fenpropathrin	Insecticide	3.NaCh	0.05	High
Fipronil	Insecticide	2.GABA	0.00811	High
Fluridone	Herbicide	F1.PDS	363	Non-toxic
Fluvalinate-tau	Varroacide	3.NaCh	4.32	Moderate
Heptachlor epoxide	Insecticide	3.NaCh	6.69	Moderate
Hexachlorobenzene	Insecticide	3.NaCh	0.468	High
Imidacloprid	Insecticide	4.nAChR	0.0398	High
Indoxacarb	Insecticide	22.NaV	147	Non-toxic
Iprodione	Fungicide	E.Sig	91.7	Low
Malathion	Insecticide	1.AChE	0.232	High
Metalaxyl	Fungicide	A.Nuc	113	Non-toxic
Methidathion	Insecticide	1.AChE	0.201	High
Methoxyfenozide	Insecticide	18.EcRs	100	Non-toxic
Metolachlor	Herbicide	K3.ICD	126	Non-toxic
Metribuzin	Herbicide	C1.PPII	56.7	Low
Myclobutanil	Fungicide	G.Sterol	161	Non-toxic
Norflurazon	Herbicide	F1.PDS	163	Non-toxic
Oxyfluorfen	Herbicide	E.PPO	100	Non-toxic
Parathion-methyl	Insecticide	1.AChE	3	Moderate
Pendimethalin	Herbicide	K3.ICD	74.9	Low
Permethrin	Insecticide	3.NaCh	0.115	High
Phosalone	Insecticide	1.AChE	4.4	Moderate
Piperonyl butoxide	Insecticide	Syn	105	Non-toxic
Potasan	Insecticide	1.AChE	5.93	Moderate
Prallethrin	Insecticide	3.NaCh	0.027	High
Pronamide	Herbicide	K1.MAI	158	Non-toxic
Propanil	Herbicide	C2.PPII	72.8	Low
Propiconazole	Fungicide	G.Sterol	67.5	Low
Pyraclostrobin	Fungicide	C.Resp	86.6	Low
Pyrethrins	Insecticide	3.NaCh	0.211	High
Pyridaben	Insecticide	21.ET	0.024	High
Pyriproxyfen	Insecticide	7.JH	87	Low
Sethoxydim	Herbicide	A.ACCase	10	Moderate
Simazine	Herbicide	C1.PPII	96.9	Low

Spiromesifen	Insecticide	23.ACC	200	Non-toxic
Tebufenozide	Insecticide	18.EcRs	234	Non-toxic
Tebuthiuron	Herbicide	C2.PPII	65	Low
Tetradifon	Insecticide	1.AChE	11	Moderate
Thiabendazole	Fungicide	B.Cyto	50	Low
Thiacloprid	Insecticide	4.nAChR	25.2	Low
THPI (Captan degradate)	Fungicide	M.Multi	135	Non-toxic
Triadimefon	Fungicide	G.Sterol	25	Low
Tribufos or DEF	Herbicide	N/A	27.1	Low
Trifloxystrobin	Fungicide	C.Resp	175	Non-toxic
Trifluralin	Herbicide	K1.MAI	68.5	Low
Vinclozolin	Fungicide	E.Sig	100	Non-toxic

[a]Mode of Action (MOA)[1]:
For ***insecticides and varroacides*** source: http://www.irac-online.org/modes-of-action/; 1.AChE – Acetylcholineasterase inhibitors; 2.GABA - GABA-gated chloride channel blockers, 3.NaCh – Sodium channel modulators, 4.nAChR -Nicotinic acetylcholine receptor competitive modulators, 7.JH – Juvenile hormone mimics, 18.EcRs - Ecdysone receptor agonists, 19.Octo - Octopamine receptor agonists, 21.ET - Mitochondrial complex I electron transport inhibitors, 22.NaV- Voltage-dependent sodium channel blockers, 23.ACC -Inhibitors of acetyl coa carboxylase, Syn – Synergist without having pesticidal activity on its own, UN- Unknown or uncertain MOA;
For ***fungicides*** source: http://www.frac.info/docs/default-source/publications/frac-code-list/frac-code-list-2016.pdf?sfvrsn=2; A.Nuc - Nucleic acids synthesis, B.Cyto - Cytoskeleton and motor proteins; C.Resp – Respiration, D.AAsyn - Amino acids and protein synthesis, E.Sig - Signal transduction, G.Sterol - Sterol biosynthesis in membranes, M.Multi - Multi-site contact activity;
For ***herbicides*** source: http://www.hracglobal.com/pages/classificationofherbicidesiteofaction.aspx; A.ACCase - Inhibition of acetyl CoA carboxylase, C1.PPII- Inhibition of photosynthesis at photosystem II (WSSA Group 5), C2.PPII -Inhibition of photosynthesis at photosystem II (WSSA Group 7), E.PPO - Inhibition of protoporphyrinogen oxidase(PPO), F1.PDS - Inhibition of carotenoid biosynthesis at the phytoene desaturase step (PDS), K1.MAI - Microtubule assembly inhibition, K3.ICD- Inhibition of cell division, N.ILS - Inhibition of lipid synthesis, N/A – not applicable
[b]Sources: US-EPA Ecotox Database http://cfpub.epa.gov/ecotox/; University of Hertfordshire (2015), The Pesticide Properties DataBase (PPDB) http://sitem.herts.ac.uk/aeru/ppdb/en/index.htm; some additional primary literature.

Table S2. Pesticide residues detected on bees (n=38) collected from the brood nest, measured as the number of detects, the pesticide load, and the HQ_{bee} score.

Pesticide	Category	Detects	HQ 50+	%	High	Low	Median	90%	95%	Mean	SEM	LOD (ppb)	Max HQ	Mean HQ	Samples above 1000 HQ
2,4 Dimethylaniline (DMA)	Varroa	1	0	2.6	275	275	275	275	275		275	50	3.55	3.55	
2,4 Dimethylphenyl formamide (DMPF)	Varroa	2	0	5.3	223	171	197	217.8	220.4	26	171	4	8.92	7.88	
Carbendazim (MBC)	Fung	1	0	2.6	14.3	14.3	14.3	14.3	14.3		14.3	5	0.29	0.29	
Chlorothalonil	Fung	1	0	2.6	35.8	35.8	35.8	35.8	35.8		35.8	5	0.32	0.32	
Coumaphos	Varroa	9	0	23.7	11	1	1.6	6.28	8.64	1.10	1	1	1.85	0.49	
DDE p,p'	Insect	1	0	2.6	6.6	6.6	6.6	6.6	6.6		6.6	2	1.03	1.03	
Dicofol	Insect	1	0	2.6	3.8	3.8	3.8	3.8	3.8		3.8	1	0.20	0.20	
Fenoxaprop-ethyl	Herb	1	0	2.6	15.4	15.4	15.4	15.4	15.4		15.4	6	0.07	0.07	
Fipronil	Insect	1	1	2.6	9.9	9.9	9.9	9.9	9.9		9.9	10	1220.7+	1220.7+	1
Fluvalinate	Varroa	31	0	81.6	172.6	1.1	9.2	91.9	129.95	8.42	1.1	1	39.95	9.07	
Methoxyfenozide	Insect	1	0	2.6	1.5	1.5	1.5	1.5	1.5		1.5	2	0.02	0.02	
Pendimethalin	Herb	2	0	5.3	27.6	25.1	26.35	27.35	27.48	1.25	25.1	6	0.37	0.35	
Pronamide	Herb	1	0	2.6	2.2	2.2	2.2	2.2	2.2		2.2	1	0.01	0.01	

HQ scores marked with + exceed 1,000 HQ, indicating that bees are at potential risk from these pesticides, as exposure is above 10% of their LD_{50} . Category of detected pesticides, number of detections, number of relevant detections (HQ 50+), pesticide prevalence (%), range of pesticide loads (high & low), median, 90th and 95th percentile of pesticide load (in ppb), mean, standard error of the mean (SEM), maximum HQ detected, mean HQ detected, and the number of samples contributing 1,000+ points to the HQ score.

Table S3. Pesticide residues detected in pooled bee bread (n=147) collected adjacent to the brood nest, measured as the number of detects, the pesticide load, and the HQ_{bee} score.

Pesticide	Category	Detects	HQ 50+	Crop with Highest Residue	%	High	Low	Median	90%	95%	Mean	SEM	LOD (ppb)	Max HQ	Mean HQ	Samples above 1000 HQ
1-Naphthol	Insect	6	2	Cran	4.1	85.5	3.6	9.1	57.2	71.4	23.3	13.0	10	193.4	52.8	
2,4 Dimethylphenyl formamide (DMPF)	Varroa	59	0	Holding	40.1	1117.0	9.1	73.2	270.6	528.6	138.3	24.5	4	44.7	5.5	
Aldicarb sulfone	Insect	3	0	Orange	2.0	97.2	43.5	59.7	89.7	93.5	66.8	15.9	2	1.2	0.8	
Aldicarb sulfoxide	Insect	12	11	Orange	8.2	1245.0	77.0	551.5	1031.3	1131.7	602.4	107.9	20	676.6	327.4	
Atrazine	Herb	20	0	Apples	13.6	37.3	5.4	10.7	28.3	31.8	13.5	2.1	6	0.4	0.1	
Azinphos methyl	Insect	3	2	Apples	2.0	107.0	3.9	69.6	99.5	103.3	60.2	30.1	6	597.8	336.1	
Azoxystrobin	Fung	40	0	Cukes	27.2	83.1	1.0	10.5	59.6	66.4	20.0	3.4	2	0.7	0.2	
Bifenthrin	Insect	8	7	Honey	5.4	5.9	1.4	3.0	5.8	5.8	3.5	0.5	1	143.2	85.9	
Captan	Fung	16	0	Cukes	10.9	832.0	25.0	78.1	442.0	550.0	169.1	55.1	10	6.2^	1.3	
Carbaryl	Insect	19	16	Cran	12.9	364.0	19.5	36.9	221.0	242.5	76.5	21.1	30	823.5	173.2	
Carbendazim (MBC)	Fung	5	0	Apples	3.4	17.4	1.5	1.8	12.4	14.9	5.5	3.0	5	0.3	0.1	
Chlorfenvinphos	Insect	1	0	Honey	0.7	10.7	10.7	10.7	10.7	10.7	10.7		6	4.6	4.6	
Chlorothalonil	Fung	87	13	Cran	59.2	26600	1.2	48.1	14564.0	18770.0	2750.6	693.3	5	239.6^	24.8^	
Chlorpyrifos	Insect	50	22	Apples	34.0	235.0	1.1	2.9	128.9	200.8	33.4	9.2	1	3084.0+	438.0	9
Coumaphos	Varroa	133	28	Holding	90.5	3260.0	1.0	10.0	531.0	806.6	174.0	36.1	1	549.7	29.3	
Coumaphos oxon	Varroa	4	0	Holding	2.7	24.1	5.8	16.1	22.3	23.2	15.5	3.8	5	4.1	2.6	
Cyfluthrin	Insect	11	10	Cukes	7.5	7.4	1.1	4.0	7.2	7.3	4.5	0.7	4	265.2	160.6	
Cyhalothrin total	Insect	6	0	Cukes	4.1	2.3	0.9	1.2	2.1	2.2	1.4	0.2	1	12.6	7.7	
Cypermethrin	Insect	8	0	Holding	5.4	7.7	1.6	3.8	6.9	7.3	4.2	0.7	4	41.0	22.2	
DDD p,p'	Insect	2	0	Cukes	1.4	13.4	11.8	12.6	13.2	13.3	12.6	0.8	4	2.1	2.0	
DDT p,p'	Insect	1	0	Honey	0.7	35.6	35.6	35.6	35.6	35.6	35.6		4	5.6	5.6	
Deltamethrin	Insect	1	1	Holding	0.7	37.5	37.5	37.5	37.5	37.5	37.5		20	864.1	864.1	
Diazinon	Insect	4	0	Cran	2.7	4.6	2.0	2.4	4.0	4.3	2.8	0.6	1	27.2	16.7	
Dicofol	Insect	11	0	Holding	7.5	88.6	4.2	16.6	53.1	70.9	27.5	7.6	1	4.8	1.5	
Diphenamid	Fung	2	0	Cukes	1.4	1.0	1.0	1.0	1.0	1.0	1.0	0.0	1	0.0	0.0	
Endosulfan I	Insect	45	0	Cukes	30.6	76.7	1.2	6.0	46.1	53.9	15.0	2.8	2	10.9	2.1	
Endosulfan II	Insect	40	0	Cukes	27.2	67.7	1.0	3.9	37.9	47.0	11.9	2.6	2	9.6	1.7	
Endosulfan sulfate	Insect	48	0	Cukes	32.7	12.6	1.0	4.3	9.7	10.5	4.7	0.5	2	0.6	0.2	

Pesticide	Category	Detects	HQ 50+	Crop with Highest Residue	%	High	Low	Median	90%	95%	Mean	SEM	LOD (ppb)	Max HQ	Mean HQ	Samples above 1000 HQ
Esfenvalerate	Insect	14	0	Cukes	9.5	5.1	1.0	3.4	4.8	5.0	3.6	0.3	2	31.5	22.4	
Famoxadone	Fung	6	0	Cukes	4.1	141.0	73.5	95.7	125.5	133.3	98.3	10.9	20	5.6^	3.9	
Fenamidone	Fung	1	0	Holding	0.7	73.9	73.9	73.9	73.9	73.9	73.9		10	0.8	0.8	
Fenhexamid	Fung	6	0	Blue	4.1	129.0	5.8	21.6	80.1	104.5	36.2	19.0	6	0.8	0.2	
Fenpropathrin	Insect	28	27	Honey	19.0	170.0	2.0	10.5	66.2	81.9	24.6	6.9	1	3400.0+	491.2	5
Fipronil	Insect	1	1	Cukes	0.7	28.5	28.5	28.5	28.5	28.5	28.5		10	3514.2+	3514.2+	1
Fluridone	Herb	1	0	Holding	0.7	5.8	5.8	5.8	5.8	5.8	5.8		10	0.0	0.0	
Fluvalinate	Varroa	147	8	Pumpkin	100.0	469.0	3.6	54.1	151.3	224.1	77.3	6.6	1	108.6	17.9	
Indoxacarb	Insect	7	0	Cukes	4.8	113.0	10.0	84.0	108.8	110.9	79.7	13.2	3	0.8	0.5	
Methidathion	Insect	2	2	Honey	1.4	18.7	13.9	16.3	18.2	18.5	16.3	2.4	10	93.0	81.1	
Methoxyfenozide	Insect	20	0	Cukes	13.6	128.0	0.4	25.4	100.2	120.4	41.0	9.3	2	1.3	0.4	
Metolachlor	Herb	13	0	Apples	8.8	31.0	6.1	10.9	23.3	27.0	13.8	2.1	6	0.2	0.1	
Metribuzin	Herb	14	0	Cukes	9.5	10.6	1.2	2.6	9.5	10.1	4.2	0.9	1	0.2	0.1	
Myclobutanil	Fung	1	0	Holding	0.7	99.8	99.8	99.8	99.8	99.8	99.8		15	0.6	0.6	
Norflurazon	Herb	15	0	Cukes	10.2	59.6	5.1	24.0	50.1	54.3	27.2	4.3	6	0.4	0.2	
Oxyfluorfen	Herb	1	0	Holding	0.7	1.8	1.8	1.8	1.8	1.8	1.8		1	0.0	0.0	
Pendimethalin	Herb	67	0	Apples	45.6	143.0	1.1	5.5	65.1	74.4	18.9	3.3	6	1.9	0.3	
Permethrin total	Insect	1	1	Holding	0.7	28.7	28.7	28.7	28.7	28.7	28.7		10	249.6	249.6	
Phosalone	Insect	1	0	Honey	0.7	31.3	31.3	31.3	31.3	31.3	31.3		10	7.1	7.1	
Potasan	Varroa	3	0	Cukes	2.0	160.0	61.5	138.0	155.6	157.8	119.8	29.9	50	27.0	20.2	
Pronamide	Herb	8	0	Cran	5.4	173.0	17.7	57.0	121.1	147.1	68.9	18.7	1	1.1	0.4	
Propanil	Herb	2	0	Holding	1.4	358.0	265.0	311.5	348.7	353.4	311.5	46.5	10	4.9	4.3	
Propiconazole	Fung	10	0	Cran	6.8	361.0	17.3	124.6	220.6	290.8	133.9	34.5	10	5.3^	2.0	
Pyridaben	Insect	5	5	Honey	3.4	26.6	10.9	19.0	25.6	26.1	18.8	3.0	1	1108.3+	785.0	2
Sethoxydim	Herb	1	0	Blue	0.7	173.0	173.0	173.0	173.0	173.0	173.0		2	17.3	17.3	
Simazine	Herb	2	0	Honey	1.4	27.1	25.9	26.5	27.0	27.0	26.5	0.6	10	0.3	0.3	
Spiromesifen	Insect	1	0	Honey	0.7	10.0	10.0	10.0	10.0	10.0	10.0		10	0.1	0.1	
Tebufenozide	Insect	1	0	Cran	0.7	58.4	58.4	58.4	58.4	58.4	58.4		5	0.2	0.2	
Tebuthiuron	Herb	1	0	Blue	0.7	11.3	11.3	11.3	11.3	11.3	11.3		2	0.2	0.2	
Thiabendazole	Fung	2	0	Orange	1.4	5.6	4.0	4.8	5.4	5.5	4.8	0.8	1	0.1	0.1	
THPI	Fung	25	0	Honey	17.0	363.0	69.0	242.0	312.0	331.6	213.9	17.2	50	2.7	1.6	

Pesticide	Category	Detects	HQ 50+	Crop with Highest Residue	%	High	Low	Median	90%	95%	Mean	SEM	LOD (ppb)	Max HQ	Mean HQ	Samples above 1000 HQ
Trifloxystrobin	Fung	3	0	Apples	2.0	264.0	3.6	16.8	214.6	239.3	94.8	84.7	1	1.5	0.5	
Trifluralin	Herb	6	0	Cukes	4.1	14.4	1.0	2.4	12.2	13.3	5.4	2.3	1	0.2	0.1	

HQ_{bbread} scores marked with + exceed 1,000 HQ, a 10% safety threshold, indicating that bees will consume more than 10% of their LD_{50} during the nursing phase of adult life if they feed on this pollen exclusively. Fungicide HQ scores marked with ^ exceed 5 HQ, a level linked with increased colony mortality in commercial beekeeping operations. Category of detected pesticides, number of detections, number of relevant detections (HQ 50+), crop in which the highest residue was detected (Blue = blueberry; Cran = cranberry, Cukes = cucumbers; Holding = holding yards; Honey = honey production), pesticide prevalence (%), range of pesticide loads (high & low), median, 90th and 95th percentile of pesticide load (in ppb), mean, standard error of the mean (SEM), maximum HQ detected, mean HQ detected, and the number of samples contributing 1,000+ points to the HQ score.

Table S4. Pesticide residues detected in wax (n = 108) collected from the brood nest, measured as the number of detects, the pesticide load, and the HQ_{wax} score.

Pesticide	Category	Detects	HQ 50+	%	High	Low	Median	90%	95%	Mean	SEM	LOD (ppb)	Max HQ	Mean HQ	Samples above 1000 HQ
2,4 Dimethylaniline (DMA)	Varroa	59	0	54.1	3820	120.0	443.0	1668.0	2466.0	751.1	106.2	50	49.3	9.7	
2,4 Dimethylphenyl formamide (DMPF)	Varroa	91	24	83.5	43000	9.2	304.0	4910.0	11320.0	2411.2	667.7	4	1720.0 +	96.4	3
3-Hydroxycarbofuran	Insect	2	3	1.8	21.1	12.4	16.8	20.2	20.7	16.8	4.4	4	183.5	145.7	
Aldicarb sulfone	Insect	15	0	13.8	49.6	18.0	27.5	45.8	48.1	31.0	2.8	2	0.6	0.4	
Aldicarb sulfoxide	Insect	22	16	20.2	649	13.4	298.5	609.2	638.8	306.6	48.0	20	352.7	166.6	
Allethrin	Insect	1	0	0.9	1.7	1.7	1.7	1.7	1.7	1.7		10	0.3	0.3	
Atrazine	Herb	27	0	24.8	19.3	1.0	5.4	15.6	16.8	7.4	1.0	6	0.2	0.1	
Azoxystrobin	Fung	23	0	21.1	22.2	1.1	5.1	8.1	8.7	5.8	0.9	2	0.2	0.1	
Bifenthrin	Insect	17	12	15.6	14	1.6	5.1	12.2	13.2	5.5	1.0	1	339.8	133.5	
Boscalid	Fung	1	0	0.9	49	49.0	49.0	49.0	49.0	49.0		4	0.3	0.3	
Carbaryl	Insect	1	0	0.9	4.5	4.5	4.5	4.5	4.5	4.5		30	10.2	10.2	
Carbendazim (MBC)	Fung	9	0	8.3	32.6	5.1	14.2	28.0	30.3	15.4	3.1	5	0.7	0.3	
Carfentrazone ethyl	Herb	1	0	0.9	4.9	4.9	4.9	4.9	4.9	4.9		1	0.1	0.1	
Chlorfenopyr	Insect	1	0	0.9	3.6	3.6	3.6	3.6	3.6	3.6		1	30.0	30.0	
Chlorferone	Insect	4	4	3.7	2830	349.0	586.0	2189.8	2509.9	507.0	585.2	50	477.2	85.5	
Chlorothalonil	Fung	75	3	68.8	53700	1.0	361.0	1772.0	3791.0	1635.0	756.9	5	483.8 ^	14.7 ^	
Chlorpyrifos	Insect	58	21	53.2	68.9	1.1	2.7	14.8	25.2	6.8	1.5	1	904.2	89.8	
Coumaphos	Varroa	109	90	100.0	20500	44.9	943.0	3874.0	5690.0	1755.7	264.9	1	3457.0 +	296.1	5
Coumaphos oxon	Varroa	108	2	99.1	638	1.3	51.5	155.3	207.7	76.5	7.9	5	107.6	12.9	
Cyfluthrin	Insect	4	4	3.7	13.8	3.2	10.5	13.7	13.7	9.5	2.5	4	494.6	340.5	
Cyhalothrin total	Insect	4	0	3.7	7.9	2.6	5.3	7.5	7.7	5.3	1.2	1	43.2	28.8	
Cypermethrin	Insect	5	4	4.6	17.8	4.5	11.2	16.0	16.9	11.4	2.2	4	94.7	60.4	

Pesticide	Category	Detects	HQ 50+	%	High	Low	Median	90%	95%	Mean	SEM	LOD (ppb)	Max HQ	Mean HQ	Samples above 1000 HQ
Cyprodinil	Fung	5	0	4.6	106	8.7	15.1	73.2	89.6	33.2	18.4	1	0.3	0.1	
DDE p,p'	Insect	1	0	0.9	30.8	30.8	30.8	30.8	30.8	30.8		2	4.8	4.8	
Deltamethrin	Insect	7	7	6.4	263	107.0	124.0	224.6	243.8	152.3	21.7	20	6059.9 +	3508.9 +	7
Diazinon	Insect	1	0	0.9	1.4	1.4	1.4	1.4	1.4	1.4		1	8.3	8.3	
Dicofol	Insect	5	0	4.6	16.2	5.7	11.1	14.7	15.5	10.2	2.0	1	0.9	0.6	
Dieldrin	Insect	2	1	1.8	12.1	6.9	9.5	11.6	11.8	9.5	2.6	10	53.3	41.9	
Dimethomorph	Fung	1	0	0.9	133	133.0	133.0	133.0	133.0	133.0		20	4.3	4.3	
Endosulfan I	Insect	62	0	56.9	16.8	1.4	4.4	10.7	12.2	5.4	0.4	2	2.4	0.8	
Endosulfan II	Insect	38	0	34.9	10.8	1.2	3.7	9.0	9.8	4.6	0.4	2	1.5	0.7	
Endosulfan sulfate	Insect	11	0	10.1	7.3	1.3	2.3	6.0	6.7	2.9	0.6	2	0.3	0.1	
Esfenvalerate	Insect	19	8	17.4	29.1	1.0	4.5	16.8	18.2	8.8	1.7	2	179.6	54.2	
Ethion	Insect	2	0	1.8	131	83.6	107.3	126.3	128.6	107.3	23.7	10	12.2	10.0	
Ethofumesate	Herb	2	0	1.8	560	224.0	392.0	526.4	543.2	392.0	168.0	5	11.2	7.8	
Fenbuconazole	Fung	8	0	7.3	75.2	7.4	39.6	58.7	66.9	37.7	7.7	10	0.5	0.3	
Fenpropathrin	Insect	36	35	33.0	74.5	2.3	16.8	50.7	58.3	23.1	3.3	1	1490.0 +	461.1	4
Fipronil	Insect	1	1	0.9	35.9	35.9	35.9	35.9	35.9	35.9		10	4426.6 +	4426.6 +	1
Fluvalinate	Varroa	109	107	100.0	28700	148.0	4310.0	9580.0	11300.0	4895.3	401.6	1	6643.5 +	1133.2 +	53
Heptachlor epoxide	Insect	1	0	0.9	13.3	13.3	13.3	13.3	13.3	13.3		10	2.0	2.0	
Hexachlorobenzene (HCB)	Fung	1	0	0.9	1	1.0	1.0	1.0	1.0	1.0		1	2.1	2.1	
Imidacloprid	Insect	2	2	1.8	13.6	2.4	8.0	12.5	13.0	8.0	5.6	1	341.7	201.0	
Iprodione	Fung	1	0	0.9	169	169.0	169.0	169.0	169.0	169.0		20	1.8	1.8	
Malathion	Insect	1	1	0.9	35.1	35.1	35.1	35.1	35.1	35.1		4	151.3	151.3	
Metalaxyl	Fung	1	0	0.9	1.4	1.4	1.4	1.4	1.4	1.4		2	0.0	0.0	
Methidathion	Insect	6	1	5.5	11.8	2.9	5.9	10.9	11.4	6.8	1.4	10	58.7	33.8	
Methoxyfenozide	Insect	33	0	30.3	495	3.5	42.3	183.0	332.6	88.6	20.1	2	5.0	0.9	
Metribuzin	Herb	1	0	0.9	8	8.0	8.0	8.0	8.0	8.0		1	0.1	0.1	

Pesticide	Category	Detects	HQ 50+	%	High	Low	Median	90%	95%	Mean	SEM	LOD (ppb)	Max HQ	Mean HQ	Samples above 1000 HQ
Norflurazon	Herb	9	0	8.3	5.1	1.1	2.7	3.7	4.4	2.6	0.4	6	0.0	0.0	
Oxyfluorfen	Herb	8	0	7.3	27.4	2.7	6.1	16.1	21.8	8.6	2.9	1	0.3	0.1	
Parathion methyl	Insect	1	0	0.9	4	4.0	4.0	4.0	4.0	4.0		2	1.3	1.3	
Pendimethalin	Herb	32	0	29.4	60	2.5	5.3	10.2	11.1	7.4	1.7	6	0.8	0.1	
Permethrin total	Insect	2	2	1.8	276	31.0	153.5	251.5	263.8	153.5	122.5	10	2400.0 +	1334.8 +	1
Piperonyl butoxide	Insect	1	0	0.9	208	208.0	208.0	208.0	208.0	208.0		6	2.0	2.0	
Prallethrin	Insect	2	2	1.8	6.8	4.3	5.6	6.6	6.7	5.6	1.3	4	251.9	205.6	
Pronamide	Herb	9	0	8.3	22.8	1.7	2.5	13.6	18.2	6.1	2.3	1	0.1	0.0	
Propiconazole	Fung	1	0	0.9	166	166.0	166.0	166.0	166.0	166.0		10	2.5	2.5	
Pyraclostrobin	Fung	5	0	4.6	13.8	1.8	11.3	13.8	13.8	9.5	2.3	2	0.2	0.1	
Pyrethrins	Insect	8	8	7.3	154	19.0	60.2	144.9	149.5	71.5	18.8	50	729.9	338.7	
Pyridaben	Insect	1	1	0.9	5.4	5.4	5.4	5.4	5.4	5.4		1	225.0	225.0	
Pyriproxyfen	Insect	2	0	1.8	7.6	2.2	4.9	7.1	7.3	4.9	2.7	1	0.1	0.1	
Tebufenozide	Insect	10	0	9.2	27.7	2.0	5.3	18.3	23.0	8.0	2.6	5	0.1	0.0	
Tetradifon	Varroa	1	0	0.9	4.7	4.7	4.7	4.7	4.7	4.7		1	0.4	0.4	
Thiabendazole	Fung	1	0	0.9	7.4	7.4	7.4	7.4	7.4	7.4		1	0.1	0.1	
Thiacloprid	Insect	4	0	3.7	7.8	1.9	5.9	7.5	7.7	5.4	1.3	1	0.3	0.2	
Triadimefon	Fung	1	0	0.9	2.4	2.4	2.4	2.4	2.4	2.4		2	0.1	0.1	
Tribufos (DEF)	Insect	1	0	0.9	7.6	7.6	7.6	7.6	7.6	7.6		2	0.3	0.3	
Trifloxystrobin	Fung	2	0	1.8	22.4	3.1	12.8	20.5	21.4	12.8	9.7	1	0.1	0.1	
Trifluralin	Herb	12	0	11.0	1.5	1.0	1.3	1.4	1.4	1.3	0.0	1	0.0	0.0	
Vinclozolin	Fung	1	0	0.9	2	2.0	2.0	2.0	2.0	2.0		1	0.0	0.0	

HQ scores marked with + exceed 1,000 HQ, a 10% safety threshold, indicating that bees will contact more than 10% of their LD_{50} during the nursing phase of adult life. Fungicide HQ scores marked with ^ exceed 5 HQ, a level linked with increased colony mortality in commercial beekeeping operations. . Category = category of detected pesticides, number of detections, number of relevant detections (HQ 50+), pesticide prevalence (%), range of pesticide loads (high & low), median, 90th and 95th percentile of pesticide load (in ppb), mean, standard error of the mean (SEM), maximum HQ detected, mean HQ detected, and the number of samples contributing 1,000+ points to the HQ score.

Table S5
Residues detected at 100+ ppb in bee bread

Catogory	Name	N
Fungicide	Captan	7
Fungicide	Chlorothalonil	35
Fungicide	Dimethomorph	1
Fungicide	Famoxadone	3
Fungicide	Fenhexamid	1
Fungicide	Iprodione	1
Fungicide	Propiconazole	6
Fungicide	THPI	22
Fungicide	Trifloxystrobin	1
	**Fungicide TOTAL**	**77**
Varroacide	DMPF	23
Varroacide	Coumaphos	30
Varroacide	Fluvalinate	39
	**Varrocide TOTAL**	**92**
Insecticide	Aldicarb sulfoxide	11
Insecticide	Azinphos methyl	1
Insecticide	Carbaryl	3
Insecticide	Chlorpyrifos	8
Insecticide	Fenpropathrin	1
Insecticide	Methoxyfenozide	2
Insecticide	Potasan	2
	**Insecticide TOTAL**	**28**
Herbicide	Pendimethalin	1
Herbicide	Pronamide	1
Herbicide	Propanil	2
Herbicide	Sethoxydim	1
	**Herbicide TOTAL**	**5**
	**Grand TOTAL**	**202**

1 Johnson, R. M., Ellis, M. D., Mullin, C. A. & Frazier, M. Pesticides and honey bee toxicity - USA. *Apidologie* **41**, 312-331, doi:10.1051/apido/2010018 (2010).
